# Genomic prediction with haplotype blocks in wheat

**DOI:** 10.3389/fpls.2023.1168547

**Published:** 2023-05-09

**Authors:** Yohannes Fekadu Difabachew, Matthias Frisch, Anna Luise Langstroff, Andreas Stahl, Benjamin Wittkop, Rod J. Snowdon, Michael Koch, Martin Kirchhoff, László Cselényi, Markus Wolf, Jutta Förster, Sven Weber, Uche Joshua Okoye, Carola Zenke-Philippi

**Affiliations:** ^1^Institute of Agronomy and Plant Breeding II, Justus Liebig University, Gießen, Germany; ^2^Institute of Agronomy and Plant Breeding I, Justus Liebig University, Gießen, Germany; ^3^Institute for Resistance Research and Stress Tolerance, Julius Kühn Institute, Quedlinburg, Germany; ^4^Deutsche Saatveredelung AG, Lippstadt, Germany; ^5^Nordsaat Saatzucht GmbH, Langenstein, Germany; ^6^Department of Cereal Breeding, W. von Borries-Eckendorf GmbH & Co. KG, Leopoldshöhe, Germany; ^7^German Seed Alliance GmbH, Holtsee, Germany; ^8^Saaten-Union Biotec GmbH, Leopoldshöhe, Germany

**Keywords:** genomic prediction, wheat, haplotype blocks, prediction accuracy, cross-validation

## Abstract

Haplotype blocks might carry additional information compared to single SNPs and have therefore been suggested for use as independent variables in genomic prediction. Studies in different species resulted in more accurate predictions than with single SNPs in some traits but not in others. In addition, it remains unclear how the blocks should be built to obtain the greatest prediction accuracies. Our objective was to compare the results of genomic prediction with different types of haplotype blocks to prediction with single SNPs in 11 traits in winter wheat. We built haplotype blocks from marker data from 361 winter wheat lines based on linkage disequilibrium, fixed SNP numbers, fixed lengths in cM and with the R package HaploBlocker. We used these blocks together with data from single-year field trials in a cross-validation study for predictions with RR-BLUP, an alternative method (RMLA) that allows for heterogeneous marker variances, and GBLUP performed with the software GVCHAP. The greatest prediction accuracies for resistance scores for *B. graminis*, *P. triticina*, and *F. graminearum* were obtained with LD-based haplotype blocks while blocks with fixed marker numbers and fixed lengths in cM resulted in the greatest prediction accuracies for plant height. Prediction accuracies of haplotype blocks built with HaploBlocker were greater than those of the other methods for protein concentration and resistances scores for *S. tritici*, *B. graminis*, and *P. striiformis*. We hypothesize that the trait-dependence is caused by properties of the haplotype blocks that have overlapping and contrasting effects on the prediction accuracy. While they might be able to capture local epistatic effects and to detect ancestral relationships better than single SNPs, prediction accuracy might be reduced by unfavorable characteristics of the design matrices in the models that are due to their multi-allelic nature.

## Highlights

- Use of haplotype blocks instead of single SNP markers leads to greater accuracy of genomic prediction of quantitative and qualitative traits in wheat.

## 1 Introduction

Haplotype blocks, most often defined as a set of adjacent markers on a chromosome, were originally proposed as a means of reducing the number of single-nucelotide polymorphisms (SNPs) required to infer the genotype of an individual by the use of tag SNPs (van den Oord and Neale, 2004). This was particularly important when genotyping costs were still very high. More recently, “haplotype stacking”, i.e. the combination of favorable haplotype blocks, has been suggested as a promising way for breeders to exploit available genetic variation (Voss-Fels et al., 2019). Moreover, haplotype blocks can identify relationship structures in breeding material and founder lines (Coffman et al., 2020). Functional haplotypes use additive and epistatic marker effects to combine SNPs into haplotype blocks, rather than combining consecutive SNPs. They were shown to identify more candidate regions in a genome-wide association study (GWAS) than single SNPs or other types of haplotype blocks (Liu et al., 2019). Other studies focused on the use of haplotype blocks in genomic prediction. Observed increases in prediction accuracy compared to single SNPs were usually attributed to either local epistasis which is by default captured by haplotype blocks (Jiang et al., 2018; Da et al., 2022) or to the fact that the LD between quantitative trait loci (QTL) and haplotype blocks might be greater than the LD between QTL and single SNPs (Hess et al., 2017). Additionally, it was argued combining SNPs into haplotype blocks can reduce the parameter space in genomic prediction by covering genome stretches that are in linkage disequilibrium (LD) (Cuyabano et al., 2014).

Studies on genomic prediction with haplotype blocks have been conducted for different species, different traits, different types of haplotype blocks and different estimation methods. Investigations have been carried out primarily in animal data sets. In six traits in sheep, prediction accuracies with a GBLUP model which used haplotype blocks were either greater than or similar to the prediction accuracies observed with SNPs only (Araujo et al., 2022). In the three carcass traits liveweight, dressing percentage, and longissimus dorsi muscle weight in beef cattle, haplotype blocks based on either LD or 5, 10, or 20 different SNPs were used in predictions together with either genomic best unbiased prediction (GBLUP) or Bayesian models. It depended on the combination of the trait, the type of haplotype blocks, and the prediction model whether prediction accuracies were greater than, similar to or smaller than the respective reference with single SNPs only (Li et al., 2022). In a Duroc population, the prediction accuracies with GBLUP models that incorporated either haplotype blocks with fixed sizes of 50 to 5000 kilobases per block or haplotype blocks based on the location of genes were up to 7.4% greater than with models that used SNPs only (Bian et al., 2021). In seven traits in humans, increases in prediction accuracies of 1.86 to 8.12% were shown for GBLUP with haplotype blocks with either fixed numbers of SNPs or fixed chromosome distances, or gene-based haplotype blocks (Liang et al., 2020). In Korean cattle, GBLUP with haplotype blocks built from either a fixed number of SNPs, a fixed length in base pairs, or agglomerative hierarchical clustering based on LD showed increased accuracy compared to SNPs for carcass weight and eye muscle area, but found small or no increases in accuracy for backfat thickness (Won et al., 2020). For three traits in three different breeds of dairy cattle, using haplotype blocks of fixed lengths in kb rather than SNPs increased prediction accuracy with different Bayesian methods, with the exception of long (> 500 kb) haplotype blocks. Moreover, increases could only be observed in some combination of traits and breeds but not in others (Hess et al., 2017). In a dairy cattle population, genomic prediction of milk protein, fertility, and mastitis was carried out with LD-based haplotype blocks and either GBLUP or a Bayesian mixture model. An average LD threshold of *D*’ > 0.45 increased the prediction accuracies for all three traits. For the other LD thresholds, it depended on the combination of trait and prediction model whether the prediction accuracies for haplotype blocks were greater than those for single SNPs (Cuyabano et al., 2014).

Fewer studies are available for genomic prediction with haplotype blocks in plants. GBLUP with haplotype blocks of 5, 10, 15, or 20 adjacent SNPs resulted in greater prediction accuracies than GBLUP with single SNPs for genomic prediction of yield, test weight, and protein content in a set of wheat lines (Sallam et al., 2020). The use of LD-based haplotype blocks in genomic prediction with different Bayesian models led to greater prediction accuracies compared to single SNPs in *Eucalyptus globulus* (Ballesta et al., 2019). In two data sets with rice genotypes and doubled-haploid maize lines, only a small subset of the traits showed an increase in prediction accuracy with GBLUP based on haplotype blocks with fixed lengths of 2 to 10 SNPs compared to GBLUP with SNP markers (Jiang et al., 2018). Genomic prediction with Bayesian methods was carried out for haplotype blocks built based on LD or with the four-gamete method in rice and maize. The use of haplotype blocks led to greater prediction accuracies in the maize breeding population while in rice, the use of single SNPs was more efficient (Matias et al., 2017).

The simplest ways of constructing haplotypes blocks is to group a fixed number of SNPs or all SNPs within a certain genetic or physical distance on the chromosome into a block. More sophisticated methods employ the LD between SNPs and built haplotype blocks out of those SNPs which are commonly inherited together, shifting the meaning of the block from distance on the chromosome to joint inheritance of SNPs within a block. Some procedures aim to exploit the haplotype diversity across genotypes and result in a haplotype block library that is representative for most of the original SNP data (Zhang et al., 2002; Pook et al., 2019). Other authors built haplotype blocks based on identified genes (Bian et al., 2021) or local genealogy (Edriss et al., 2013).

For wheat, results for genomic prediction with haplotype blocks are available only for blocks with a fixed number of SNPs (Sallam et al., 2020). Our goal was to compare the accuracy of genomic prediction with haplotype blocks to the standard prediction with single SNPs in 11 traits in winter wheat. In particular, our objectives were to compare (1) different types of block-building methods, (2) different prediction models, and (3) the interaction between both with each other and to a baseline scenario (GBLUP with single SNP markers).

## 2 Materials and methods

### 2.1 Field data

378 elite wheat lines were evaluated in a one-year field trial in 2020. We evaluated the resistances against *Septoria tritici*, *Fusarium graminearum*, *Puccinia triticina* (brown rust), *Blumeria graminis* (mildew), and *Puccinia striiformis* (yellow rust). Resistances were scored in observation plots in one replication at one (*S. tritici*, *F. graminearum*), two (*P. triticina*), or three locations (*B. graminis*, *P. striiformis*). In case there was more than one location, the arithmetic mean of the two or three observations was used as the resistance score. In order to improve the readability of the manuscript, we use only the name of the disease instead of the full term for the trait, *e.g.* “*S. tritici*” instead of “*S. tritici* resistance score”.

A p-rep design with 54 genotypes in the second replication was conducted at six locations in Germany (Asendorf, Niedersachsen; Böhnshausen, Sachsen-Anhalt; Granskevitz, Mecklenburg-Vorpommern; Groß-Gerau, Hessen; Hovedissen, Nordrhein-Westfalen; Leutewitz, Sachsen) and one location in Poland (Gola) for the quantitative traits grain yield, protein concentration, starch concentration, hectoliter weight, and plant height. Results from Böhnshausen and Groß-Gerau were removed from the analysis due to extreme weather conditions. The remaining field data were analyzed with the mixed linear model


g=μ+l+e+l:e+r:e+b:r:e+ϵ

where *l* is the effect of the line, *e* is the effect of the environment (location), *l*:*e* is the genotype-by-environment interaction, *r*:*e* is the replication-within-environment effect, *b*:*r*:*e* is the block effect nested within replication and environment, and ϵ is the residual. The genotype was analyzed as a fixed factor, the remaining factors of the model were random. The adjusted entry means were used in further calculations. Protein yield was calculated as the product of yield and protein concentration.

### 2.2 Genotypic data

All wheat lines were genotyped with the 25k Illumina iSelect SNP array (SGS TraitGenetics, Gatersleben, Germany). All SNP markers with more than two recorded alleles, more than 10% missing values and an expected heterozygosity of < 5% as well as all individuals with more than 10% missing marker information were excluded from the analysis. As a result, 16,667 SNP markers and 361 genotypes remained for further analysis. We used this data set for all further calculations.

### 2.3 Methods for building haplotype blocks

Haplotype blocks were built with the following methods:

LD-AVERAGE-0, LD-AVERAGE-1, LD-FLANKING-0, LD-FLANKING-1: LD-based haplotype blocks were based on *r*^2^ as a measure of LD (Zhao et al., 2005). *r*^2^ was calculated between all SNPs on each chromosome. Haplotype blocks were then built based on different threshold values *t* = 0.1, 0.2, 0.3, 0.4, 0.5, 0.6, 0.7, 0.8, and 0.9 for *r*^2^. For methods LD-AVERAGE-0 and LD-AVERAGE-1, *t* was compared to the new average LD between each of the marker pairs within the block if a new SNP was added. For methods LD-FLANKING-0 and LD-FLANKING-1, *t* was compared to the LD between the new SNP and the SNP flanking the block. In each case, the new SNP was added if the threshold *t* was exceeded. Blocks were built for tolerance values of zero (all SNPs in the block must meet the criterion; LD-AVERAGE-0 and LD-FLANKING-0) or one (one SNP in the block may fail to meet the criterion; LD-AVERAGE-1 and LD-FLANKING-1). SNPs that could not be assigned to any block were treated as haplotype blocks with just one SNP. An example for how LD-based haplotype blocks were built can be found in **Supplementary File S2**.

FIXED-SNP: *n* = 5, 10, 20, 50, or 100 adjacent SNPs were grouped into haplotype blocks. An example for how haplotype blocks were built with method FIXED-SNP can be found in **Supplementary File S2**.

FIXED-CM: Adjacent SNPs within window sizes of 5, 10, or 20 cM were grouped into haplotype blocks.

HAPLOBLOCKER: The R package HaploBlocker (Pook et al., 2019) starts with haplotype blocks built from windows with a fixed number of SNPs. These blocks are then clustered and merged. Blocks are identified, filtered and extended in an iterative procedure (Pook et al., 2019). The result is a library of blocks that are most representative of the data set. Each block can be either present or absent in each genotype but no variants are defined. In the default setting, overlapping blocks are possible. The percentage of the SNP markers that is covered by the blocks in the final haplotype block library is the target coverage (Pook et al., 2019). We used different combinations of a target coverage of 0.90 or 0.95, a starting window size of 5 or 12 SNPs and either overlapping or non-overlapping blocks (**Table 1**).

**Table 1:** Methods for building haplotype blocks with method HAPLOBLOCKER and statistics of the resulting haplotype blocks.

	Version							
	HB1	HB2	HB3	HB4	HB5	HB6	HB7	HB8
Window size	5	5	5	5	12	12	12	12
Target coverage	0.90	0.90	0.95	0.95	0.90	0.90	0.95	0.95
Overlapping blocks	no	yes	no	yes	no	yes	no	yes
Haplotype blocks	5,818	4,725	8,239	7,612	7,594	7,753	7,967	12,499
SNPs per block								
Average	8	24	7	23	13	37	13	37
Maximum	95	308	85	411	132	471	96	471
Distinct variants per block								
Average	1	1	1	1	1	1	1	1
Maximum	1	1	1	1	1	1	1	1

Additionally, we investigated subsets of the marker data with *n* = 500, 1,000, 2,000, 3,000, 4,000, 5,000, 6,000, 7,000, 8,000, 9,000, 10,000, 11,000, and 12,000 randomly selected SNPs. A different set of *n* SNPs was used in each cross-validation run.

### 2.4 Genomic prediction of marker and haplotype block effects

We used ridge regression best linear unbiased prediction (RR-BLUP) of marker and haplotype block effects (Meuwissen et al., 2001), which was technically implemented using a transformation to an animal model (Shen et al., 2013). It was chosen as a baseline scenario since it has proved to be relatively robust in many circumstances (VanRaden, 2008; Clark and Werf, 2013). In order to get robust results for singular design matrices that may occur during the simulation replications we used method 2 of Nazarian and Gezan (2016). The method is available in our software package SelectionTools (http://population-genetics.uni-giessen.de/software0/). For comparison, we used estimation of the error and genetic variance components with restricted maximum likelihood and partitioning according to ANOVA variance components (RMLA) (Hofheinz and Frisch, 2014) which allows for heterogeneous marker variances.

Both RR-BLUP and RMLA are based on the assumption of bi-allelic SNPs. Since haplotype blocks are multi-allelic by nature, we had to re-parametrize the marker matrices to allow for the application of both methods, resulting in a design matrix **Z** with one column per haplotype block variant (*cf.* Jiang et al. (2018); Hess et al. (2017); Matias et al. (2017); Cuyabano et al. (2014); Villumsen et al. (2009)). An example can be found in **Supplementary File S2**. Haplotype blocks built with method HAPLOBLOCKER are encoded as either present or absent and do not have variants. The resulting presence-absence matrix for the blocks was treated as a re-parametrized marker matrix in these cases.

We used the software GVCHAP (Prakapenka et al., 2020) for a multi-allelic haplotype model (Da, 2015) which performs genomic best linear unbiased prediction (GBLUP) with a genomic additive relationship matrix **Z***_GVCHAP_* based on haplotype blocks. **Z***_GVCHAP_* is the design matrix **Z** from above, transposed, scaled (Hayes and Goddard, 2010), multiplied by -1 and with one column eliminated for a “reference variant” for each of the haplotype blocks. The mixed linear model that was then used for GBLUP included additive effects for the haplotype blocks only (Model 4 in Prakapenka et al. (2020)). GVCHAP was not used for haplotype blocks built with method HAPLOBLOCKER.

### 2.5 Assessment of prediction accuracy

Haplotype blocks were built based on the complete data set with 361 genotypes. Cross-validation was then employed in order to assess the prediction accuracy. In each of 1000 cross-validation runs, the data set was randomly divided into a training set with 289 genotypes and a validation set with 72 genotypes. The same splits into training and validation set were used for all the sets of predictors. The prediction accuracy *r*(*y,ŷ*) was calculated as the correlation between the actual phenotypic values *y* and the predicted phenotypic values *ŷ* in the validation set.

### 2.6 Software

We used R version 4.0.3 for all calculations. The adjusted entry means of the genotypes were estimated with ASReml-R 4.1.0.110. Haplotype blocks were built with either the R package SelectionTools version 22.1 or with HaploBlocker version 1.6.06. RR-BLUP and RMLA were calculated with SelectionTools version 22.1. GBLUP was calculated with GVCHAP version 2.1.

## 3 Results

### 3.1 Statistics of haplotype blocks built with different methods

For LD-based blocks with an average *r*^2^ of at least 0.1 between all SNPs within a block and zero tolerance (method LD-AVERAGE-0), 210 haplotype blocks with an average number of 79 SNPs and an average of 186 variants were identified (**Table 2**). The greatest number of SNPs in one block was 671, the greatest number of variants was 360. 177 SNPs remained unassigned so that the total number of haplotype blocks and unassigned SNPs was 387. When the threshold was raised to 0.9, 2,214 haplotype blocks with an average of 3 SNPs and 17 variants were identified. The maximum numbers were 21 SNPs and 19 variants per block. With 9,694 unassigned SNPs, the total number of haplotype blocks and unassigned SNPs was 11,908 (**Table 2**).

**Table 2:** Statistics of haplotype blocks (with ≥2 SNPs) built based on linkage disequilibrium (LD).

	LD threshold								
	0.1	0.2	0.3	0.4	0.5	0.6	0.7	0.8	0.9
LD-AVERAGE-0									
Haplotype blocks	210	639	1,160	1,680	2,100	2,333	2,443	2,414	2,214
Unassigned SNPs	177	898	2,022	3,329	4,515	5,673	6,757	7,971	9,694
Total	387	1,537	3,182	5,009	6,615	8,006	9,200	10,385	11,908
SNPs per block									
Average	79	25	13	8	6	5	4	4	3
Maximum	671	260	150	93	82	57	44	31	21
Distinct variants per block									
Average	186	72	35	22	17	15	15	15	17
Maximum	360	354	303	236	140	78	64	36	19
LD-AVERAGE-1									
Haplotype blocks	204	611	1,085	1,583	2,012	2,268	2,403	2,400	2,199
Unassigned SNPs	171	803	1,789	2,928	4,185	5,478	6,609	7,895	9,632
Total	375	1,414	2,874	4,511	6,197	7,746	9,012	10,295	11,831
SNPs per block									
Average	81	26	14	9	6	5	4	4	3
Maximum	671	252	150	95	82	66	44	31	21
Distinct variants per block									
Average	190	75	38	23	18	16	15	15	18
Maximum	360	354	303	264	246	161	64	36	19
LD-FLANKING-0									
Haplotype blocks	2,252	2,611	2,784	2,836	2,828	2,795	2,749	2,603	2,294
Unassigned SNPs	3,324	4,215	4,911	5,525	6,061	6,716	7,431	8,404	10,020
Total	5,576	6,826	7,695	8,361	8,889	9,511	10,180	11,007	12,314
SNPs per block									
Average	6	5	4	4	4	4	3	3	3
Maximum	67	45	32	32	32	28	28	21	18
Distinct variants per block									
Average	15	13	13	13	13	13	13	14	17
Maximum	87	64	45	39	38	36	36	20	15
LD-FLANKING-1									
Haplotype blocks	1,363	1,769	2,032	2,187	2,262	2,314	2,346	2,275	2,056
Unassigned SNPs	1,556	2,250	2,926	3,634	4,152	4,991	5,819	6,933	8,759
Total	2,919	4,019	4,958	5,821	6,414	7,305	8,165	9,208	10,815
SNPs per block									
Average	11	8	7	6	6	5	5	4	4
Maximum	123	76	76	64	56	56	56	34	34
Distinct variants per block									
Average	29	21	18	16	16	15	15	16	18
Maximum	242	168	150	109	106	91	99	82	32

For methods LD-AVERAGE-0 and LD-AVERAGE-1, the average *r^2^* between all SNPs within a haplotype block including the new SNP is compared to the LD threshold. For methods LD-FLANKING-0 and LD-FLANKING-1, *r^2^* between the new SNP and the SNP flanking a haplotype block is compared to the LD threshold. Either all SNPs within a block must fulfill the criterion (LD-AVERAGE-0, LD-FLANKING-0) or there may be one SNP that does not fulfill the criterion (LD-AVERAGE-1, LD-FLANKING-1

A tolerance level of one (method LD-AVERAGE-1) which allows for one SNP per block that does not fulfill the criterion changed these numbers only slightly (**Table 2**). For an average *r*^2^ of at least 0.1 between all SNPs within a block, 204 haplotype blocks with an average number of 81 SNPs and an average of 190 variants were found. The maximum numbers were 671 SNPs and 360 variants per block. Since 171 SNPs remained unassigned, the total number of haplotype blocks and unassigned SNPs was 375. When the threshold was raised to 0.9, 2,199 haplotype blocks with an average of 3 SNPs and 18 variants were identified. At most, there were 21 SNPs and 19 variants per block, and 9,632 SNPs remained unassigned (**Table 2**).

The influence of the tolerance parameter was much greater for methods LD-FLANKING-0 and LD-FLANKING-1 in which the LD of the new SNP with the SNP flanking the block is compared to the threshold value. Also, the blocks for comparable *r*^2^ thresholds were much smaller than those for methods LD-AVERAGE-0 and LD-AVERAGE-1 (**Table 2**).

With a tolerance of zero (method LD-FLANKING-0) and an *r*^2^ threshold value of 0.1, 2,252 haplotype blocks with an average number of 6 SNPs and an average of 15 variants were identified (**Table 2**). The maximum values were 67 SNPs and 87 variants per block and 3,324 SNPs remained unassigned, resulting in a total number of 5,576 haplotype blocks and unassigned SNPs. With an *r*^2^ threshold of 0.9, 2,294 haplotype blocks were found. They had 3 SNPs and 17 variants on average and 18 SNPs and 15 variants maximum. Together with 10,020 unassigned SNPs, there was a total of 12,314 haplotype blocks and unassigned SNPs (**Table 2**).

When one SNP per block was allowed to not exceed the threshold value (method LD-FLANKING-1), 1,363 haplotype blocks with an average number of 11 SNPs and 29 variants were identified for an *r*^2^ threshold of 0.1 (**Table 2**). The greatest number of SNPs in one block was 123, the greatest number of variants was 242. Since 1,556 SNPs remained unassigned, there were 2,919 haplotype blocks and unassigned SNPs total. When the threshold was raised to 0.9, 2,056 haplotype blocks with an average of 4 SNPs and 18 variants were identified. The maximum numbers were 34 SNPs and 32 variants per block, and 8,759 SNPs remained unassigned so that the total number of haplotype blocks and unassigned SNPs was 10,815 (**Table 2**).

For a fixed block size of *n* = 5 SNPs (method FIXED-SNP), 3,339 haplotype blocks were found with an average of 14 variants and a maximum of 47 variants (**Table 3**). The number of haplotype blocks reduced to 178 for a block size of *n* = 100 SNPs. The average variant number was 262, the maximum number was 356 (**Table 3**). For method FIXED-CM, The number of haplotype blocks reduced from 1,400 to 535 when the window size of each block increased from 5 to 20 cM. On average, there were 12 and 31 SNPs and 35 and 98 variants per block, respectively. The maximum numbers were 100 SNPs and 248 variants for blocks with a length of 5 cM and 272 SNPs and 350 variants for blocks with a length of 20 cM (**Table 3**). For both methods, FIXED-SNP and FIXED-CM, there were almost no unassigned SNPs (**Table 3**).

**Table 3:** Statistics of haplotype blocks built based on a fixed number of SNPs per haplotype block (FIXED-SNP) or a fixed window size in cM (FIXED-CM).

	FIXED-SNP					FIXED-CM		
	Number of SNPs per block					Window size in cM		
	5	10	20	50	100	5	10	20
Haplotype blocks	3,339	1,676	843	344	178	1,400	897	535
Unassigned SNPs	4	3	2	0	0	0	1	1
Total	3,343	1,679	845	344	178	1,400	898	536
SNPs per block								
Average	5	10	20	48	94	12	19	31
Maximum	5	10	20	50	100	100	165	272
Distinct variants per block								
Average	14	31	71	172	262	35	58	98
Maximum	47	177	311	348	356	248	318	350

Each haplotype block consists of ≥2 SNPs.

Blocks built with method HAPLOBLOCKER (Pook et al., 2019) varied in three parameters: the starting window size (5 or 12 SNPs), the target coverage of the final haplotype block library (0.90 or 0.95), and the possibility for overlapping blocks (yes or no) (**Table 1**). The number of haplotype blocks was between 4,725 and 12,499 across all investigated versions (**Table 1**). When overlapping blocks were allowed, the number of haplotype blocks was always smaller than with non-overlapping blocks while the average and maximum numbers of SNPs per block were greater. The number of haplotype blocks was also greater with a greater target coverage and with a starting window size of 12 SNPs when compared to the alternative version with all the other parameters constant. The target coverage did not influence the average and maximum numbers of variants per block. Both numbers were greater for a starting window size of 12 (**Table 1**).

### 3.2 Genomic prediction with a reduced set of SNPs

RR-BLUP with reduced SNP numbers achieved the same level of prediction accuracy as the full set of SNPs with at least 3,000 (yield, *B. graminis*), 4,000 (*S. tritici*, *P. striiformis*, *F. graminearum*), 5,000 (protein concentration, protein yield, starch concentration, hectoliter weight, *P. triticina*), or 6,000 (plant height) SNPs, respectively (**Supplementary Figure S1**).

### 3.3 RR-BLUP with haplotype blocks

**Figure 1:**
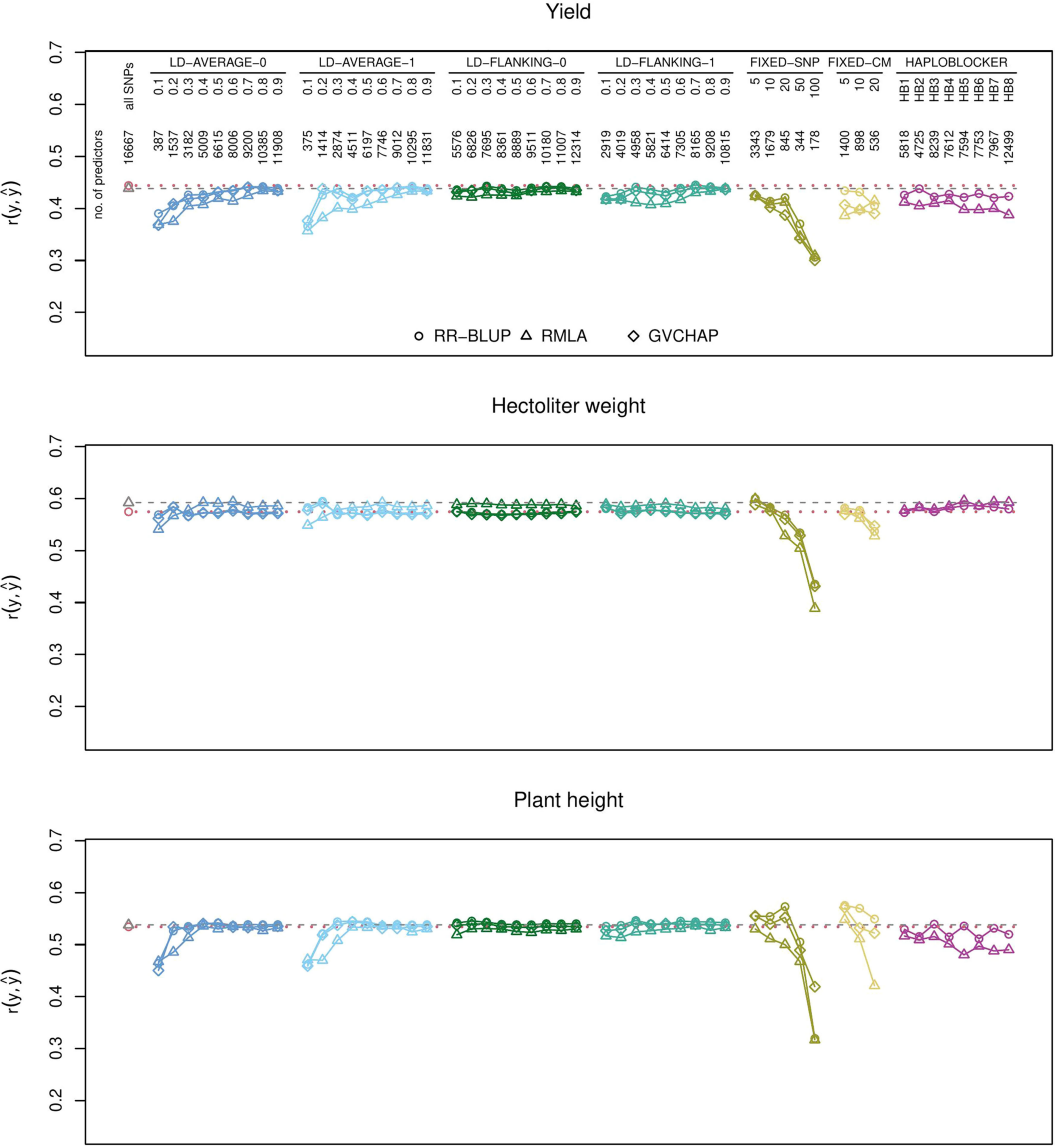
Prediction accuracies for genomic prediction of yield, hectoliter weight, and plant height with different types of haplotype blocks and estimation methods. The plots show the medians of the correlations *r*(*y*,*ŷ*) between the observed phenotypic values *y* and the predicted phenotypic values *ŷ* in the validation set for 1000 cross-validation runs. Haplotype blocks were built based on linkage disequilibrium (LD-AVERAGE-0, LD-AVERAGE-1, LD-FLANKING-0, LD-FLANKING-1) with different threshold values *t*=0.1,0.2,…0.9 for *r*^2^, with fixed numbers of SNPs per block (FIXED-SNP), with a fixed block length in cM (FIXED-CM), or with the R package HaploBlocker (HAPLOBLOCKER). Red dotted lines: Quartiles from RR-BLUP with 16,667 SNPs (baseline). Gray dashed lines: Quartiles from RMLA with 16,667 SNPs. The number of predictors is the combined number of haplotype blocks and unassigned SNPs.

**Figure 2:**
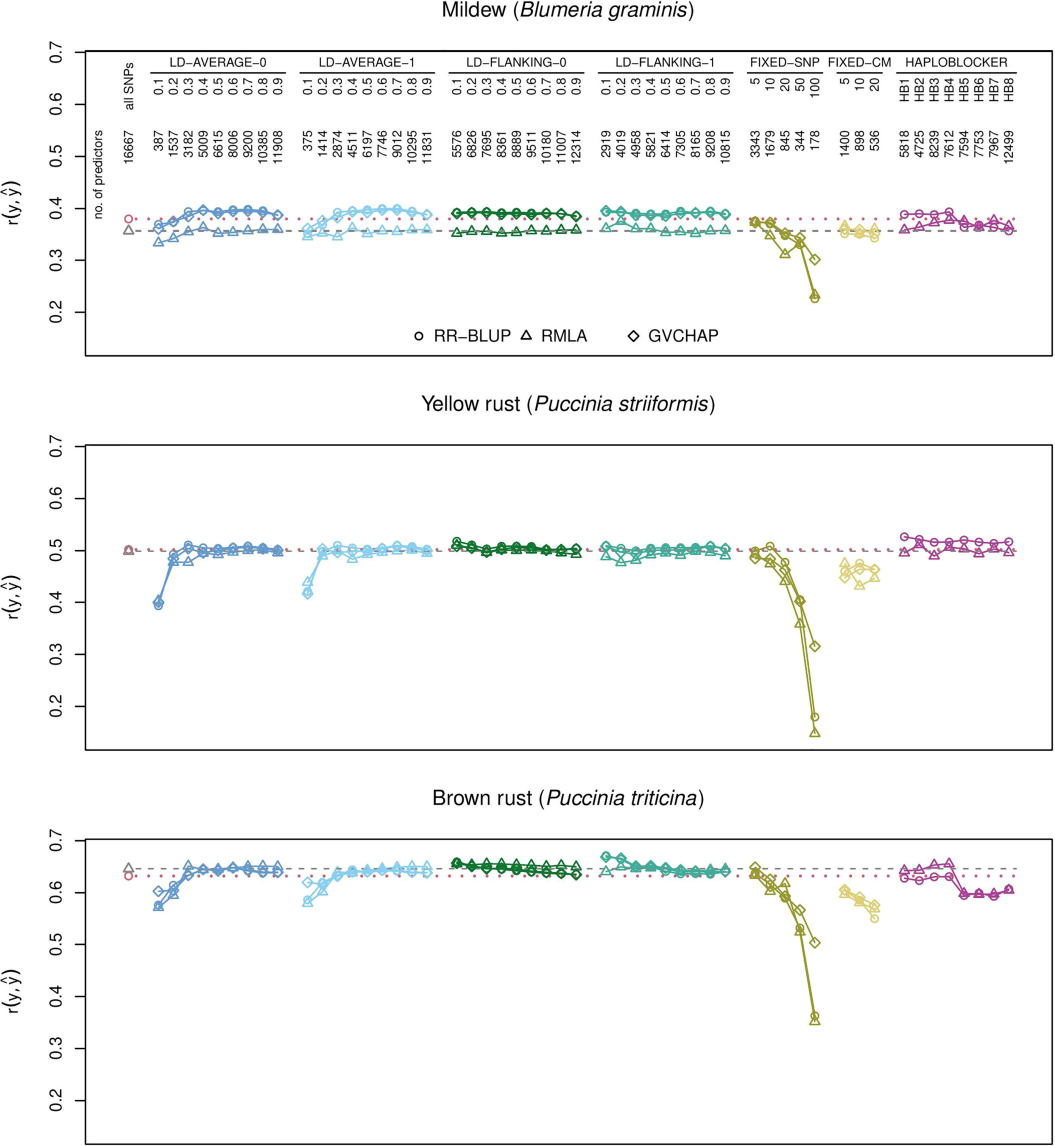
Prediction accuracies for genomic prediction of resistance scores for *B*. *graminis*, *P. striiformis*, and *P. triticina* with different types of haplotype blocks and estimation methods. The plots show the medians of the correlations *r*(*y*,*ŷ*) between the observed phenotypic values *y* and the predicted phenotypic values *ŷ* in the validation set for 1000 cross-validation runs. Haplotype blocks were built based on linkage disequilibrium (LD-AVERAGE-0, LD-AVERAGE-1, LD-FLANKING-0, LD-FLANKING-1) with different threshold values *t*=0.1,0.2,…,0.9 for *r*^2^, with fixed numbers of SNPs per block (FIXED-SNP), with a fixed block length in cM (FIXED-CM), or with the R package HaploBlocker (HAPLOBLOCKER). Red dotted lines: Quartiles from RR-BLUP with 16,667 SNPs (baseline). Gray dashed lines: Quartiles from RMLA with 16,667 SNPs. The number of predictors is the combined number of haplotype blocks and unassigned SNPs.

RR-BLUP with the full set of SNPs (baseline) had greater prediction accuracies than all block-based predictions for yield (**Figure 1**), protein yield, and starch concentration (**Supplementary Figures S4, S5**). For all other traits, there was at least one type of haplotype block for which the prediction accuracies were greater than the baseline (**Supplementary Figures S2, S3, S6–S12**). Yield, plant height and hectoliter weight and the resistance scores for *B. graminis*, *P. striiformis*, and *P. triticina* were chosen as illustrative examples for quantitative and qualitative traits, respectively (**Figures 1**, **2**). Results for all investigated traits can be found in the **Supplementary Figures S2–S12**.

Haplotype blocks with 5, 10, or 20 SNPs (method FIXED-SNP) or with a fixed length of 5, 10, or 20 cM (method FIXED-CM) showed prediction accuracies above baseline for plant height (**Figure 1**). Haplotype blocks with a fixed number of 5 SNPs and some block types built with method HAPLOBLOCKER resulted in prediction accuracies above baseline for hectoliter weight (**Figure 1**). In the case of *B. graminis*, LD-based haplotype blocks led to greater prediction accuracies than single SNPs. This was the case for all haplotype blocks built with methods LD-FLANKING-0 and LD-FLANKING-1 and for those blocks built with methods LD-AVERAGE-0 and LD-AVERAGE-1 and an LD threshold of *r*^2^ > 0.3. In this trait, haplotype blocks built with method HAPLOBLOCKER based on a starting window size of 5 SNPs also led to greater prediction accuracies compared to the baseline (**Figure 2**).

In *P. striiformis*, all haplotype blocks types built with method HAPLOBLOCKER resulted in greater prediction accuracies than single SNPs. Prediction accuracies for LD-based haplotype blocks were only greater than the baseline when the haplotype blocks were built with methods LD-FLANKING-0 or LD-FLANKING-1 and a low LD threshold of *r*^2^ > 0.1 or 0.2, which was also the case for *P. triticina* (**Figure 2**).

Some patterns were observed independent of the trait. For LD-based haplotype blocks built with methods LD-AVERAGE-0 and LD-AVERAGE-1, *r*^2^ had to be at least 0.2 or 0.3 to lead to meaningful predictions. Similarly, prediction accuracies declined considerably for haplotype blocks with a fixed SNP number of 50 or sometimes 20 (method FIXED-SNP), and a length of more than 10 or 20 cM (method FIXED-CM). For LD-based haplotype blocks built with methods LD-FLANKING-0 and LD-FLANKING-1, the influence of the threshold value on the prediction accuracies was smaller than for methods LD-AVERAGE-0 and LD-AVERAGE-1 (**Figures 1**, **2**).

Within the haplotype blocks built with method HAPLOBLOCKER, different patterns became apparent. In yield and *P. striiformis*, all prediction accuracies were on the same level (**Figures 1**, **2**). In plant height, the non-overlapping blocks resulted in greater prediction accuracies than the overlapping blocks with the same parameters (**Figure 1**). In *B. graminis* and *P. triticina*, haplotype blocks based on a starting window size of 5 SNPs showed greater prediction accuracies than haplotype blocks based on a starting window size of 12, regardless of the other parameters (**Figure 2**).

### 3.4 Alternative genomic prediction methods

It was dependent on the trait whether ridge regression with homogeneous (RR-BLUP) or heterogeneous (RMLA) marker variances resulted in greater prediction accuracies. In *B. graminis*, RR-BLUP showed greater prediction accuracies than RMLA (**Figure 2**). RR-BLUP and RMLA resulted in similar prediction accuracies for yield, plant height, and *P. striiformis* (**Figures 1**, **2**). RMLA showed greater prediction accuracies than RR-BLUP in *P. triticina* and hectoliter weight (**Figures 1**, **2**).

Prediction accuracies obtained with GBLUP with GVCHAP were mostly similar to those with RR-BLUP (**Figures 1**, **2**). The only exceptions were the haplotype blocks with a fixed number of 50 or 100 SNPs (method FIXED-SNP) and sometimes fixed block lengths of 10 or 20 cM (method FIXED-CM) where GBLUP with GVCHAP showed greater prediction accuracies than the other estimation methods (**Figures 1**, **2**). These were also the only instances in which the overall ranking of the estimation methods changed. In all other cases, the ranking of the methods remained the same across all types of haplotype blocks (**Figures 1**, **2**).

## 4 Discussion

### 4.1 Genomic prediction with haplotype blocks

#### 4.1.1 Number of haplotype blocks

The different procedures for building haplotype blocks resulted in vastly varying number of haplotype blocks and unassigned SNPs that were used for the predictions (**Tables 1**–**3**). We determined the minimum number of SNPs required for accurate predictions in order to determine whether changes in prediction accuracy were related to the coverage of the genome with SNPs. Across all traits, RR-BLUP with 3,000 through 6,000 SNPs randomly selected SNPs resulted in prediction accuracies comparable to that of the full set of 16,667 SNPs (baseline) (**Supplementary Figure S1**). It has to be noted that the number of haplotype blocks does not directly translate into the dimensions of the design matrices in the mixed linear models. In RR-BLUP with *m* bi-allelic SNP markers, the design matrix **Z** for the genotypic effects has *m* columns. The number of columns of the design matrix **Z** in a haplotype model depends on (a) the number of haplotype blocks and unassigned SNPs, and (b) the number of variants at each haplotype block. We found that very often, even though there were fewer haplotype blocks than there were single SNPs in the full marker data set, the dimensions of the resulting design matrix **Z** in the reparameterized case were roughly the same because of the high number of variants at some of the haplotype blocks. These dimensions were reduced when monomorphic block-variant combinations were eliminated in the training set. For example, building LD-based blocks with methods LD-FLANKING-0 and an *r*^2^ threshold of 0.1, 2,252 haplotype blocks and 3,324 unassigned SNPs were identified. They translated in a design matrix **Z** with 18,330 columns (block/SNP-variant combinations), which were reduced to 13,803 in the first cross-validation run after eliminating those block-variant combinations with an expected heterozygosity of less than 5% in the training set. These observations show that haplotype blocks do not reduce the parameter space, as has been claimed to be the case if they are used in genomic selection (Cuyabano et al., 2014).

In all cases in which the block-building methods resulted in less than 500 haplotype blocks and unassigned SNPs, for example with LD-based blocks built with methods LD-AVERAGE-0 and LD-AVERAGE-0 and low LD thresholds or with method FIXED-SNP and 50 or 100 SNP markers per block, the prediction accuracies were much smaller than in the baseline scenario (**Figures 1**, **2**). Conversely, 536–898 haplotype blocks and unassigned SNPs led to prediction accuracies above baseline for plant height when haplotype blocks were built with methods FIXED-SNP and FIXED-CM (**Figure 1**), an increase which could not be achieved by simply reducing the number of SNPs in the predictions (**Supplementary Figure S1**). These findings confirm that haplotype blocks do more than just eliminate noise in the form of redundant information from the SNP data since that reduction should also be achievable by a simple reduction in the number of SNPs used for the predictions.

Some authors claim that the additional information carried by haplotype blocks is mainly local epistatis (Jiang et al., 2018; Da et al., 2022). Other hypotheses include that ancestral relationships might be detected better by haplotype blocks and that the LD between causal mutations and haplotype variants might be greater than for single SNP markers (Hess et al., 2017). It is possible that this additional information content is outweighed by the properties of the design matrices because the large number of columns that belong to the variants of a single haplotype block might introduce multicollinearity and estimation errors (Matias et al., 2017). A further decrease might be caused by the substantial number of haplotype block variants that is removed when filtering out monomorphic loci.

#### 4.1.2 Block-building methods

It depended on the trait whether adding SNPs based on the average LD in the block (methods LD-AVERAGE-0 and LD-AVERAGE-1) or based on their LD with the flanking SNP of a block (methods LD-FLANKING-0 and LD-FLANKING-1) resulted in greater prediction accuracies (**Figures 1**, **2**). Similarly, allowing for no (tolerance zero) or one (tolerance one) SNP in each block that does not fulfill the LD threshold made in a difference in some cases while in others it did not. For example, there was no difference between the prediction accuracies of haplotype blocks built with methods LD-AVERAGE-0, LD-AVERAGE-1, LD-FLANKING-0, and LD-FLANKING-1 in the prediction of plant height (**Figure 1**). In *B. graminis* and *P. striiformis*, prediction accuracies for haplotype blocks built with methods LD-AVERAGE-0 and LD-AVERAGE-1 increased with an increase in the LD threshold while they decreased for haplotype blocks built with methods LD-FLANKING-0 and LD-FLANKING-1. In *P. triticina*, haplotype blocks built with method LD-FLANKING-1 resulted in much greater prediction accuracies than single SNPs while this increase was not observed for haplotype blocks built with the other methods (**Figure 2**). Overall, thresholds for *r*^2^ of 0.4 for haplotype blocks built with methods LD-FLANKING-0 and LD-FLANKING-1 and 0.6 for haplotype blocks built with methods LD-AVERAGE-0 and LD-AVERAGE-1 led to prediction accuracies comparable to that of the baseline, indicating that the information content was similar (**Figures 1**; **2**, **Supplementary Figure S1**). These haplotype blocks did not lead to greater prediction accuracies than single SNPs in any of the cases.

Prediction with haplotype blocks with a fixed number of SNPs (method FIXED-SNP) resulted in a decrease in prediction accuracies in most traits (**Figures 1**, **2**). The most notable exception was plant height which showed an increase in prediction accuracy for RR-BLUP (**Figure 1**). For the other traits, predictions of *P. striiformis*, *P. triticina*, and hectoliter weight could be increased compared to the baseline with either one or several types of haplotypes blocks built with method FIXED-SNP (**Figures 1**, **2**). The same was observed for haplotypes blocks built with method FIXED-CM: The prediction accuracies for plant height were substantially greater (**Figure 1**). In the other traits, prediction accuracies for haplotype blocks built with methods FIXED-SNP or FIXED-CM were smaller than those for haplotype blocks built based on LD (**Figures 1**, **2**). A possible reason for this finding could be that blocks with a fixed number of SNPs or fixed window size combine SNPs arbitrarily while LD-based blocks take into account information from the data set regarding the recombination frequencies. This is reflected in the finding that a relatively high number of SNPs remains unassigned with the LD-based block-building methods (**Table 2**). It can therefore be expected that LD-based blocks should capture more or less the same information about QTL for the trait even with low LD thresholds while marker-trait associations might be broken for blocks with a fixed number of SNPs or fixed length in cM.

Prediction accuracies increased slightly for *B. graminis*, *P. striiformis*, and *P. triticina* for haplotype blocks built with method HAPLOBLOCKER. For plant height, prediction accuracies for non-overlapping haplotype blocks were always greater than their counterparts with overlapping blocks, even though none of the versions led to prediction accuracies greater than RR-BLUP with the full set of SNPs. For *B. graminis* and *P. triticina*, the greatest differences within the haplotype blocks built with method HAPLOBLOCKER were between the starting window sizes 5 and 12. Prediction accuracies were comparable to that of single SNP markers for window size 5 but much smaller for window size 12 (**Figure 2**).

Trait-dependence of prediction accuracies with different block-building methods was also observed in pigs (Bian et al., 2021), humans (Liang et al., 2020), cattle (Cuyabano et al., 2014; Won et al., 2020), eucalyptus (Ballesta et al., 2019), wheat (Sallam et al., 2020) and rice and maize (Matias et al., 2017). Other authors studied the optimal haplotype block length required for estimation of the genomic relationship matrix and also arrived at the conclusion that it depends on the trait which block length is best (Ferdosi et al., 2016). Apparently, there is no single method that can generally be recommended for building meaningful blocks. If, as proposed by some authors (Jiang et al., 2018; Da et al., 2022), greater prediction accuracies are mostly due to local epistasis that is captured by the haplotype blocks, these findings raise the question if the optimal choice of haplotype blocks depends on the exact structure of “local” epistatis exhibited for the trait.

It is also possible that the genetic architecture of the trait influenced whether haplotype blocks led to greater prediction accuracies than single markers. In our data set, we observed greater prediction accuracies for haplotype blocks than for single SNPs in the prediction of oligogenic traits like the resistance scores for *B. graminis*, *P. striiformis*, and *P. triticina*. In contrast, there was no obvious and consistent advantage of using haplotype blocks for the prediction of polygenic traits like yield, hectoliter weight, and plant height (**Figures 1**, **2**).

Haplotype blocks divide the chromosome into segments and effects are then assigned to these chromosome segments rather than distributed over many markers. This approach might be beneficial for the prediction of oligogenic traits because it reduces the noise caused by a great number of markers that are not linked to genes that are causal for the trait. Additionally, haplotype blocks with a positive effect can then be used to combine favorable chromosome stretches *via* haplotype stacking (Voss-Fels et al., 2019). For highly polygenic traits, grouping markers into chromosome segments and assigning effects to segments rather than to single markers is not expected to lead to greater prediction accuracies because is it precisely the distribution of effects over many markers that corresponds to their polygenic nature. The effect of the haplotype block would then be a “net effect” that is roughly equal to the sum of the effects of the single markers in this block, not adding any additional or removing redundant information.

### 4.2 Investigating alternative genomic prediction methods

#### 4.2.1 Assumption of heterogeneous marker variances

Using a the RMLA model with heterogeneous marker variances (Hofheinz and Frisch, 2014) instead of the baseline (RR-BLUP with homogeneous marker variances) with all available SNP markers resulted in increases of prediction accuracies for *P. triticina* and hectoliter weight, decreases in prediction accuracies for *B. graminis* and equal prediction accuracies for all other traits (**Figures 1**, **2**). We had hypothesized that RMLA might be beneficial particularly for resistance traits which tend to be oligogenic rather than polygenic and might therefore benefit from the modeling of marker effects with heterogeneous variances. However, the possible advantage in the estimation of more accurate marker effects (Hofheinz and Frisch, 2014) did not translate into greater prediction accuracies for most of the traits we investigated. In most cases, prediction accuracies for genomic prediction with RMLA were either smaller than the corresponding version with RR-BLUP or the same. The overall tendencies (decreases or increases within a particular block-building method) were roughly the same as for RR-BLUP but the deviations from RR-BLUP were greater than those with GVCHAP. Greater *r*^2^ thresholds for the LD-based haplotype blocks were required for RMLA to obtain the same prediction accuracies for plant height as RR-BLUP (**Figure 1**). It depended on the trait whether the influence of the estimation method, as shown for eucalyptus (Ballesta et al., 2019), or the influence of the block building method on the prediction accuracies was greater (**Figures 1**, **2**). We cannot make a general recommendation for the use of either RR-BLUP or RMLA for the investigated traits.

#### 4.2.2 Multi-allelic GBLUP with GVCHAP

The main difference between RR-BLUP with SelectionTools and GBLUP with GVCHAP is the construction of the genomic relationship matrix G. In SelectionTools, **G**=**ZZ**’ with **Z** the re-parametrized design matrix. **Z** is subjected to several transformations, including centering with the allele frequencies, to arrive a realized relationship **G** in GVCHAP (Da, 2015). Results for RR-BLUP and GVCHAP were very similar with the exception of the long haplotype blocks built with a fixed number of *n *= 50 or 100 SNPs or with a fixed length of 10 or 20 cM (**Figures 1**, **2**). In these special cases, GVCHAP showed greater prediction accuracies than RR-BLUP even though the prediction accuracies were smaller than for RR-BLUP with 16,667 SNPs. The genomic relationship that is captured by both methods is apparently mostly the same and a difference arises only when the blocks become relatively long (**Figures 1**, **2**, **Table 2**). These instances were also the only ones in which the ranking of the prediction methods (RR-BLUP, RMLA, GBLUP with GVCHAP) changed. In all other cases, their ranking remained the same over all types of haplotype blocks used for the predictions (**Figures 1**, **2**).

### 4.3 Conclusions

Prediction accuracies for most traits in our data set were greater when haplotype blocks were used instead of single SNP markers in genomic prediction. The ranking of the block-building methods was trait-dependent, with some methods leading to greater prediction accuracies than single SNPs in some traits and to smaller prediction accuracies in others. The greatest prediction accuracies for resistance scores for *B. graminis*, *P. triticina*, and *F. graminearum* were obtained with LD-based haplotype blocks while blocks with fixed marker numbers and fixed lengths in cM resulted in the greatest prediction accuracies for plant height. Prediction accuracies of haplotype blocks built with the R package HaploBlocker (Pook et al., 2019) were greater than those of the other methods for protein concentration and resistances scores for *S. tritici*, *B. graminis*, and *P. striiformis*. For the resistance scores for *B. graminis*, prediction accuracies were greater than for standard RR-BLUP if marker variances were assumed to be heterogeneous (Hofheinz and Frisch, 2014). Results for multi-allelic prediction with software GVCHAP (Prakapenka et al., 2020) were similar to those from RR-BLUP in most cases. The dependence of prediction accuracies on trait and estimation method was also observed in other studies (Ballesta et al., 2019; Liang et al., 2020; Sallam et al., 2020; Won et al., 2020; Bian et al., 2021). It is important to note that all these studies used different species, blocking methods, and marker effect estimation procedures and do not allow for direct numerical comparison of the results. Nevertheless, their findings support our conclusions that (1) haplotype blocks have the potential to increase the accuracy of genomic prediction in winter wheat, and (2) the choice of the best block-building method is trait-dependent. It is likely that the trait-dependence is caused by properties of the haplotype blocks that have overlapping and contrasting effects on the prediction accuracy. While they might be able to capture local epistatic effects and to detect ancestral relationships better than single SNPs, prediction accuracy might be reduced by unfavorable characteristics of the design matrices in the models that are due to their multi-allelic nature. Additionally, haplotype blocks might be better suited for the prediction of oligogenic than polygenic traits. In oligogenic traits like resistances, they might improve the correct assignment of effects to the underlying genes, while in polygenic traits, the precision of marker effect estimates cannot be improved. Our results suggest that building haplotype blocks allows efficient haplotype stacking for oligogenic resistances in wheat.

## Data availability statement

The datasets presented in this study can be found in online repositories. The names of the repository/repositories and accession number(s) can be found below: https://github.com/czp-jlu/haploblocks, czp-jlu/haploblocks.

## Author contributions

MF, RS, and AS conceived the study. MKo, MKi, LC, MW, and JF collected the field data and genotypic data. AL, AS, BW, and MF evaluated the field data. YD, UO, and CZ-P carried out the genomic prediction. YD and CZ-P wrote the manuscript. SW contributed to writing the manuscript. All authors contributed to the article and approved the submitted version.
