# Supplementary material for: Genomic prediction with haplotype blocks in wheat
Source: Front Plant Sci. 2023 May 9;14:1168547. doi: 10.3389/fpls.2023.1168547 (PMC10203549; doi:10.3389/fpls.2023.1168547)
Supplement: Supplementary file 1 [file DataSheet_1.pdf]

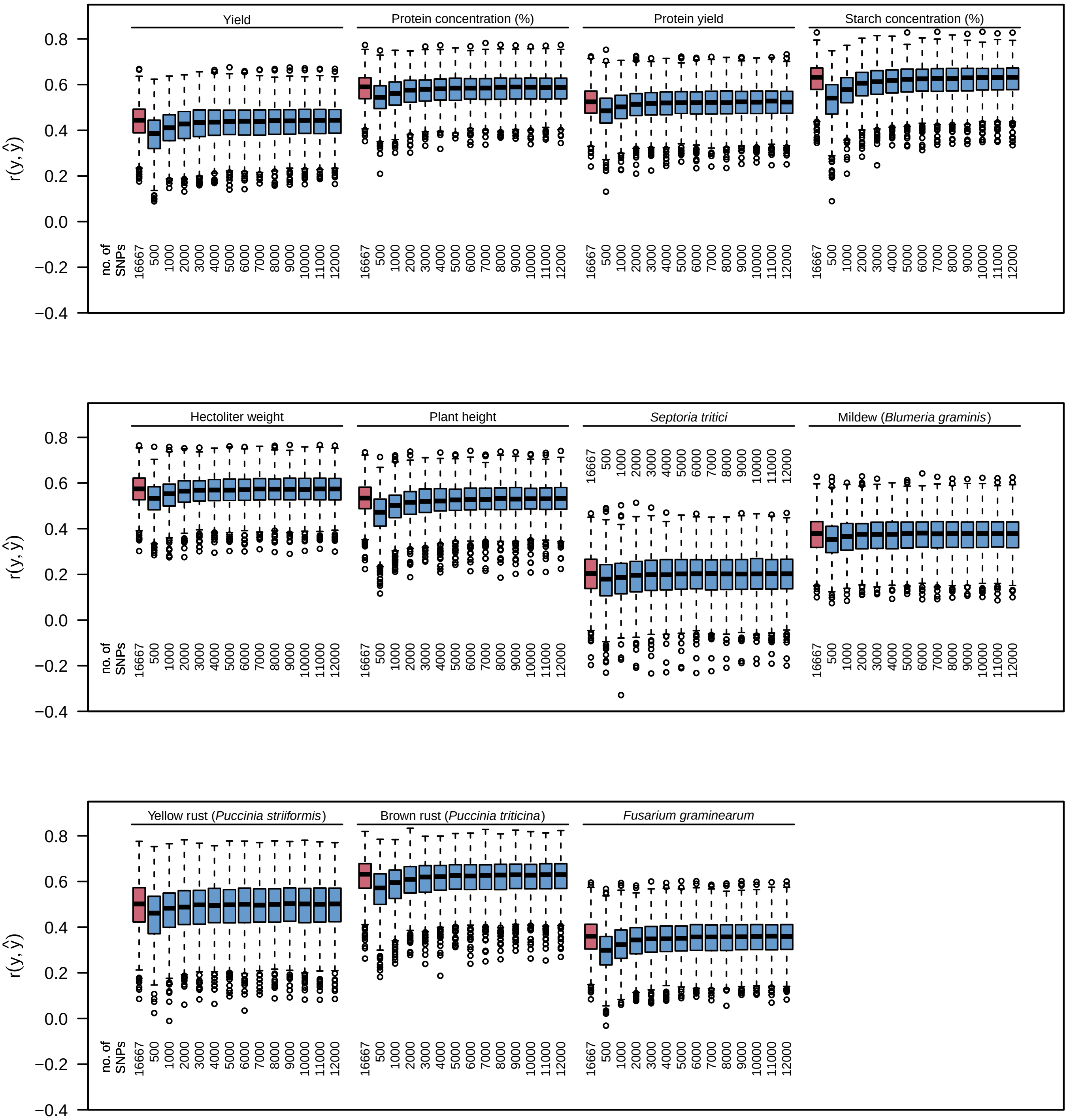

Figure S1. Prediction accuracies for genomic prediction of eleven traits in winter wheat with different numbers of SNP markers. Marker effects were estimated with RR-BLUP. The boxplots show the correlations $r(y, \hat{y})$ between the observed phenotypic values y and the predicted phenotypic values $\hat{y}$ in the validation set for 1000 cross-validation runs.

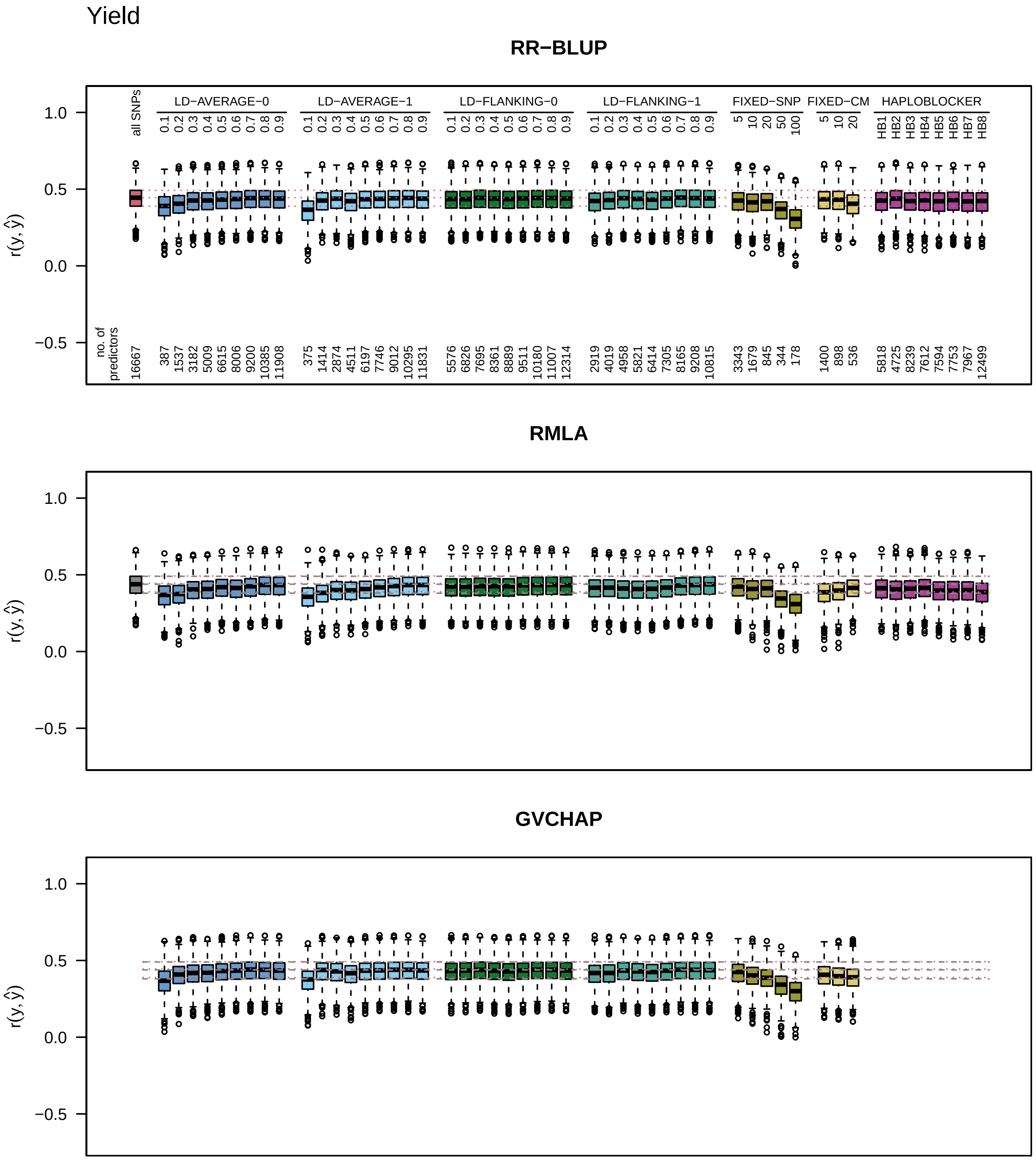

Figure S2. Prediction accuracies for genomic prediction of yield with different types of haplotype blocks and estimation methods. The boxplots show the correlations $r(y, \hat{y})$ between the observed phenotypic values y and the predicted phenotypic values $\hat{y}$ in the validation set for 1000 cross-validation runs. Haplotype blocks were built based on linkage disequilibrium (`LD-AVERAGE-0`, `LD-AVERAGE-1`, `LD-FLANKING-0`, `LD-FLANKING-1`) with different threshold values $t = 0.1, 0.2, ..., 0.9$ for r^2, with fixed numbers of SNPs per block (`FIXED-SNP`), with a fixed block length in cM (`FIXED-CM`), or with the R package HaploBlocker (`HAPLOBLOCKER`). Red dotted lines: Quartiles from RR-BLUP with 16,667 SNPs (baseline). Gray dashed lines: Quartiles from RMLA with 16,667 SNPs. The number of predictors is the combined number of haplotype blocks and unassigned SNPs.

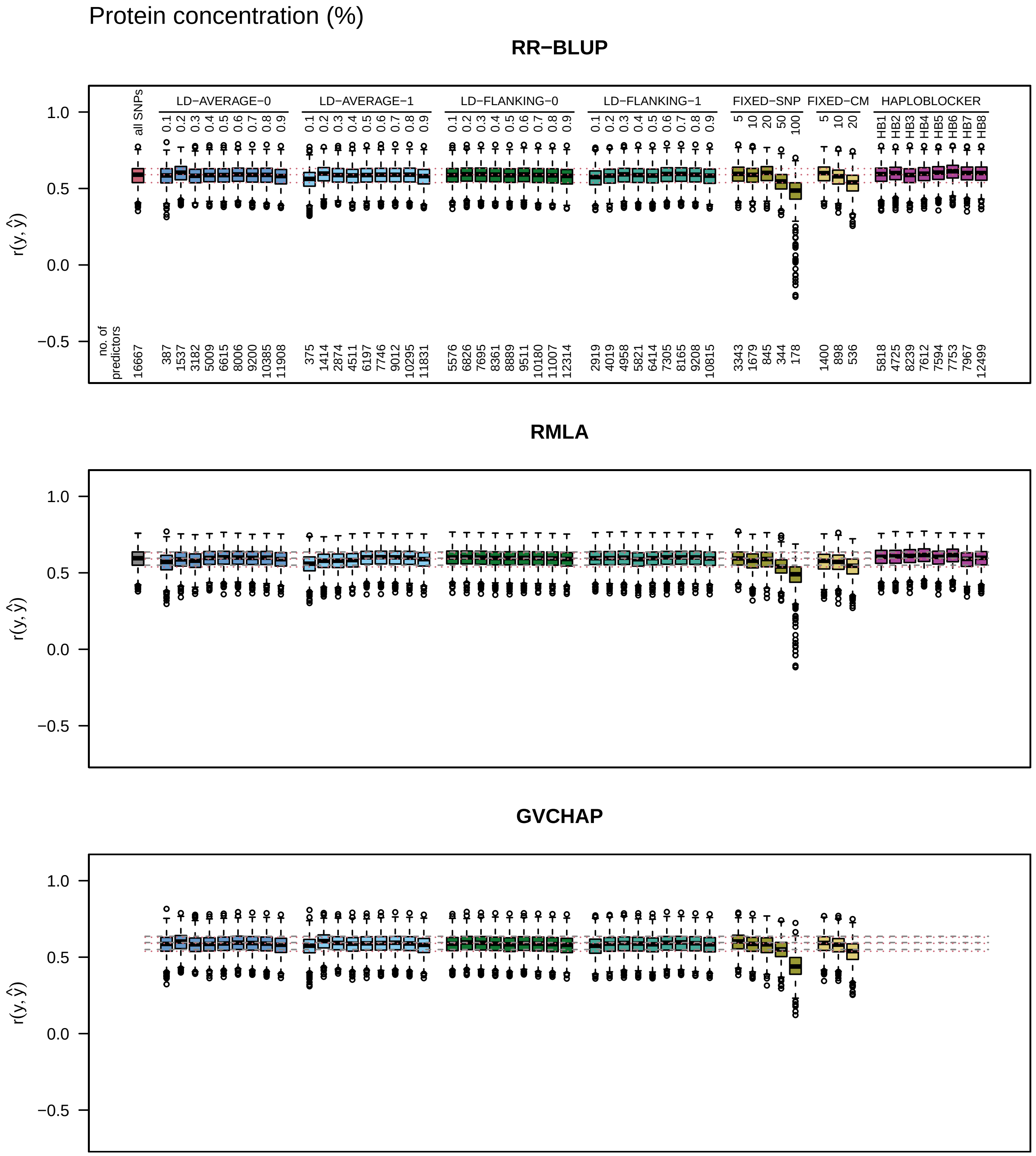

Figure S3. Prediction accuracies for genomic prediction of protein concentration with different types of haplotype blocks and estimation methods. The boxplots show the correlations $r(y, \hat{y})$ between the observed phenotypic values y and the predicted phenotypic values $\hat{y}$ in the validation set for 1000 cross-validation runs. Haplotype blocks were built based on linkage disequilibrium (`LD-AVERAGE-0`, `LD-AVERAGE-1`, `LD-FLANKING-0`, `LD-FLANKING-1`) with different threshold values $t = 0.1, 0.2, ..., 0.9$ for r^2, with fixed numbers of SNPs per block (`FIXED-SNP`), with a fixed block length in cM (`FIXED-CM`), or with the R package HaploBlocker (`HAPLOBLOCKER`). Red dotted lines: Quartiles from RR-BLUP with 16,667 SNPs (baseline). Gray dashed lines: Quartiles from RMLA with 16,667 SNPs. The number of predictors is the combined number of haplotype blocks and unassigned SNPs.

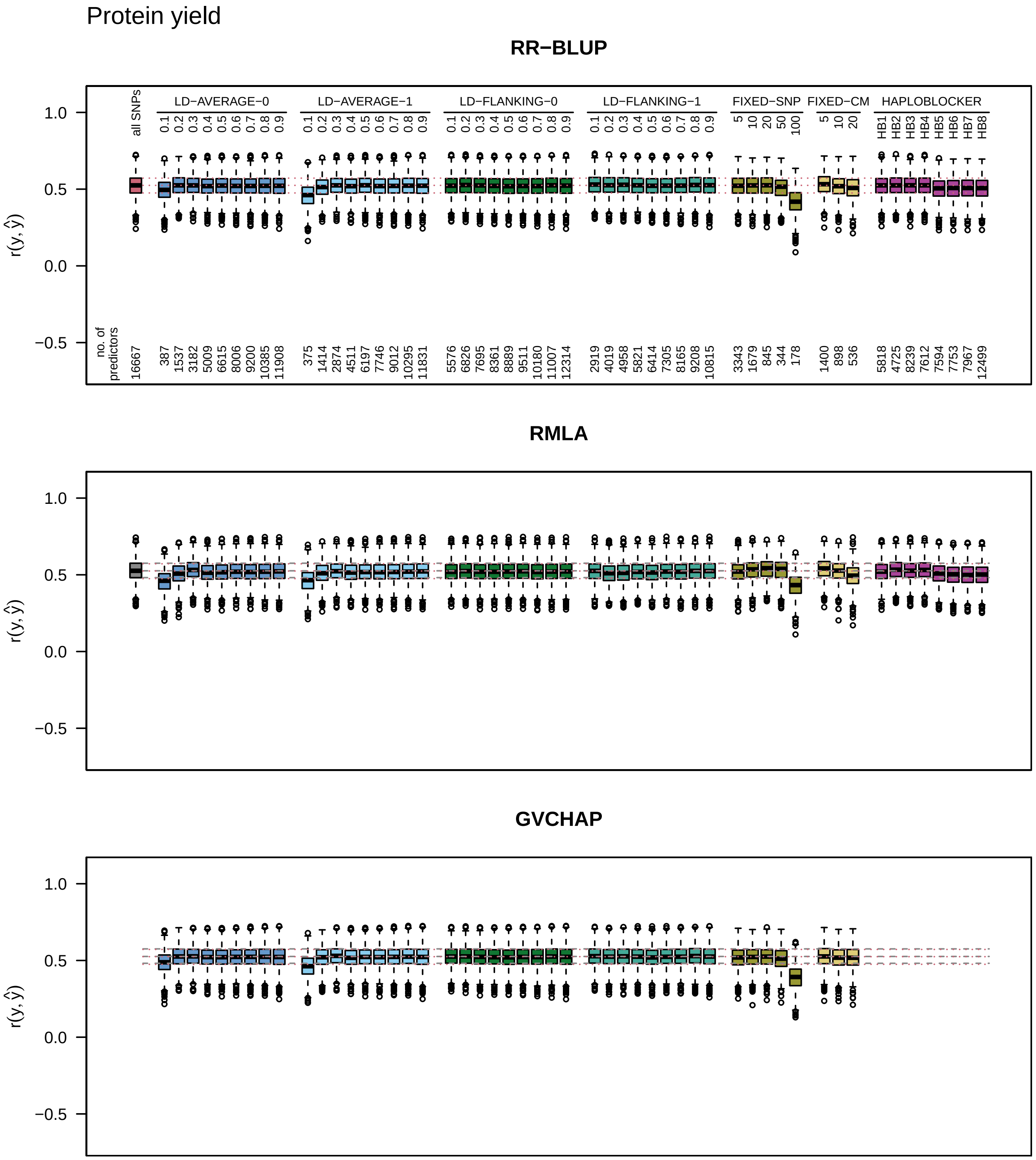

Figure S4. Prediction accuracies for genomic prediction of protein yield with different types of haplotype blocks and estimation methods. The boxplots show the correlations $r(y, \hat{y})$ between the observed phenotypic values y and the predicted phenotypic values $\hat{y}$ in the validation set for 1000 cross-validation runs. Haplotype blocks were built based on linkage disequilibrium (`LD-AVERAGE-0`, `LD-AVERAGE-1`, `LD-FLANKING-0`, `LD-FLANKING-1`) with different threshold values $t = 0.1, 0.2, ..., 0.9$ for r^2, with fixed numbers of SNPs per block (`FIXED-SNP`), with a fixed block length in cM (`FIXED-CM`), or with the R package HaploBlocker (`HAPLOBLOCKER`). Red dotted lines: Quartiles from RR-BLUP with 16,667 SNPs (baseline). Gray dashed lines: Quartiles from RMLA with 16,667 SNPs. The number of predictors is the combined number of haplotype blocks and unassigned SNPs.

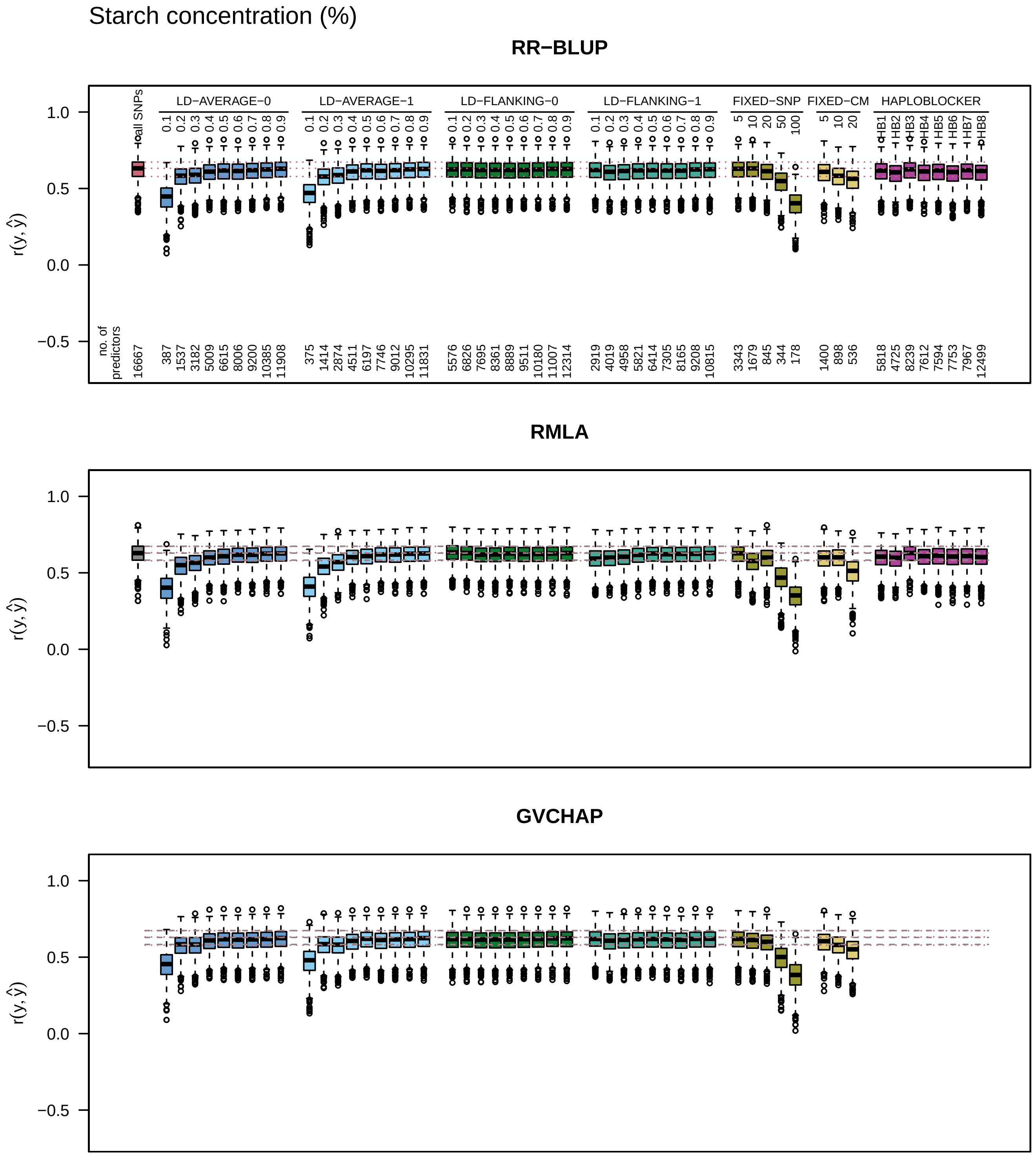

Figure S5. Prediction accuracies for genomic prediction of starch concentration with different types of haplotype blocks and estimation methods. The boxplots show the correlations $r(y, \hat{y})$ between the observed phenotypic values y and the predicted phenotypic values $\hat{y}$ in the validation set for 1000 cross-validation runs. Haplotype blocks were built based on linkage disequilibrium (LD−AVERAGE−0, LD−AVERAGE−1, LD−FLANKING−0, LD−FLANKING−1) with different threshold values $t = 0.1, 0.2, ..., 0.9$ for r^2, with fixed numbers of SNPs per block (FIXED−SNP), with a fixed block length in cM (FIXED−CM), or with the R package HaploBlocker (HAPLOBLOCKER). Red dotted lines: Quartiles from RR-BLUP with 16,667 SNPs (baseline). Gray dashed lines: Quartiles from RMLA with 16,667 SNPs. The number of predictors is the combined number of haplotype blocks and unassigned SNPs.

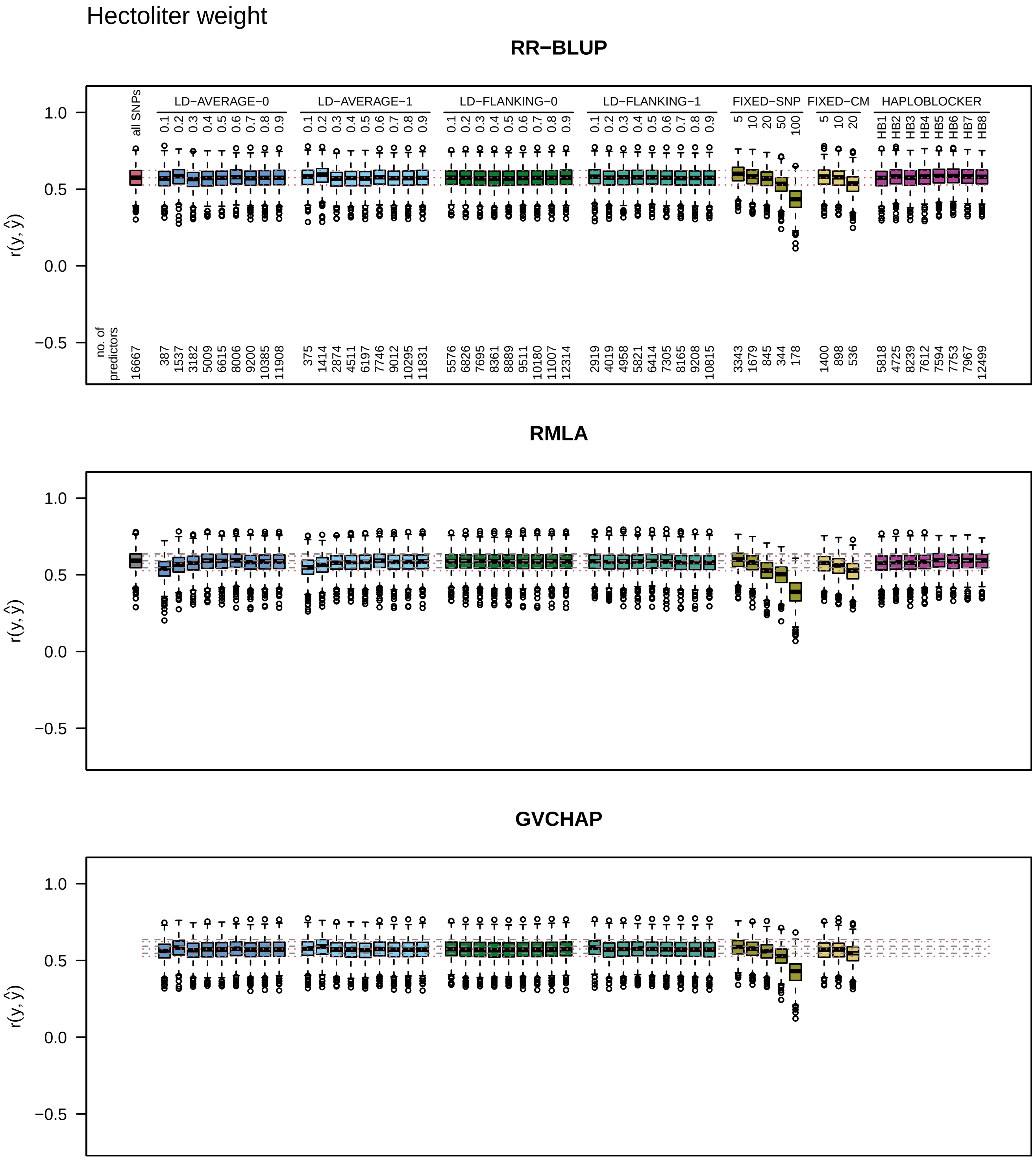

Figure S6. Prediction accuracies for genomic prediction of hectoliter weight with different types of haplotype blocks and estimation methods. The boxplots show the correlations $r(y, \hat{y})$ between the observed phenotypic values y and the predicted phenotypic values $\hat{y}$ in the validation set for 1000 cross-validation runs. Haplotype blocks were built based on linkage disequilibrium (`LD-AVERAGE-0`, `LD-AVERAGE-1`, `LD-FLANKING-0`, `LD-FLANKING-1`) with different threshold values $t = 0.1, 0.2, ..., 0.9$ for r^2, with fixed numbers of SNPs per block (`FIXED-SNP`), with a fixed block length in cM (`FIXED-CM`), or with the R package HaploBlocker (`HAPLOBLOCKER`). Red dotted lines: Quartiles from RR-BLUP with 16,667 SNPs (baseline). Gray dashed lines: Quartiles from RMLA with 16,667 SNPs. The number of predictors is the combined number of haplotype blocks and unassigned SNPs.

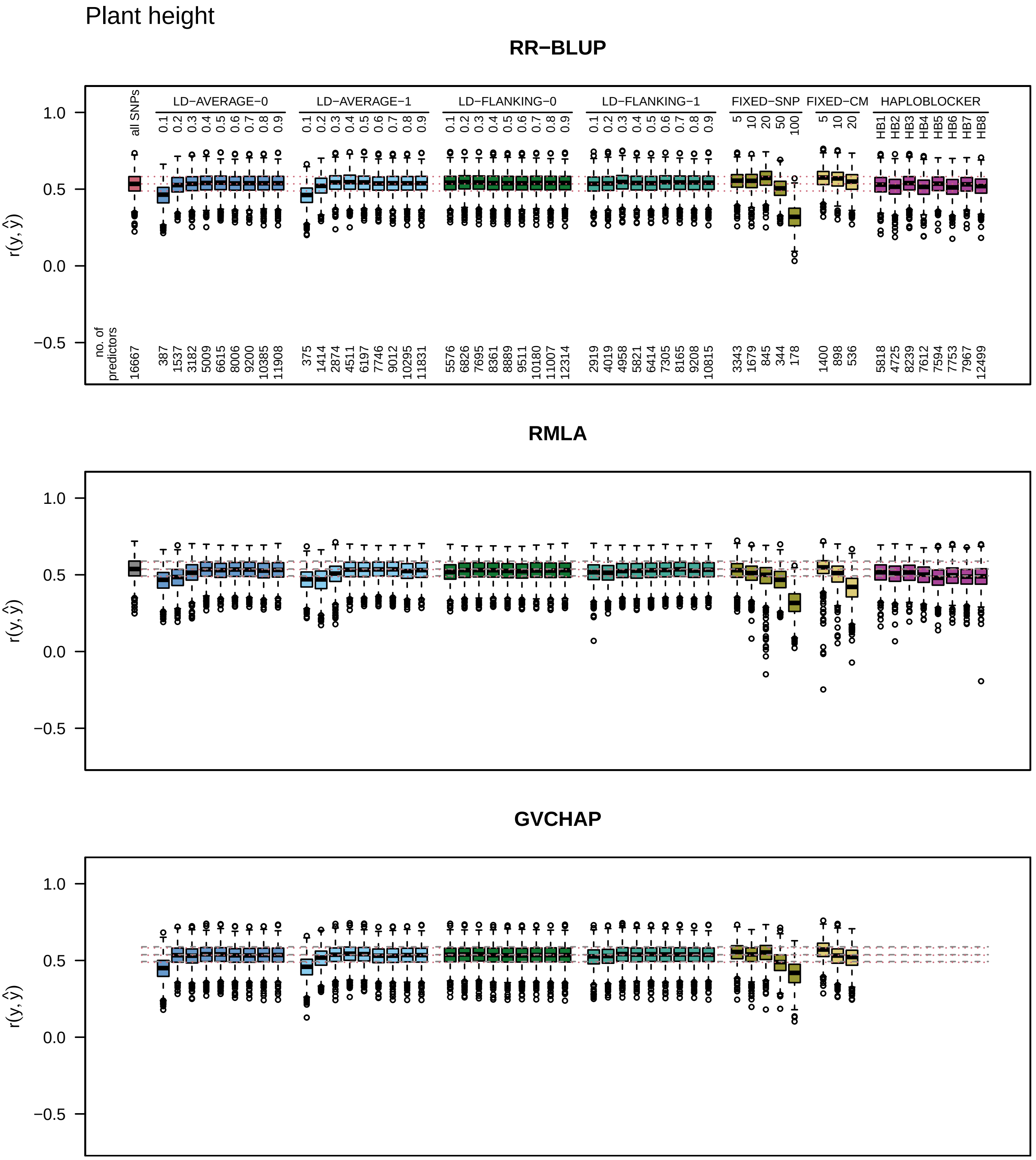

Figure S7. Prediction accuracies for genomic prediction of plant height with different types of haplotype blocks and estimation methods. The boxplots show the correlations $r(y, \hat{y})$ between the observed phenotypic values y and the predicted phenotypic values $\hat{y}$ in the validation set for 1000 cross-validation runs. Haplotype blocks were built based on linkage disequilibrium (LD−AVERAGE−0, LD−AVERAGE−1, LD−FLANKING−0, LD−FLANKING−1) with different threshold values $t = 0.1, 0.2, ..., 0.9$ for r^2, with fixed numbers of SNPs per block (FIXED−SNP), with a fixed block length in cM (FIXED−CM), or with the R package HaploBlocker (HAPLOBLOCKER). Red dotted lines: Quartiles from RR-BLUP with 16,667 SNPs (baseline). Gray dashed lines: Quartiles from RMLA with 16,667 SNPs. The number of predictors is the combined number of haplotype blocks and unassigned SNPs.

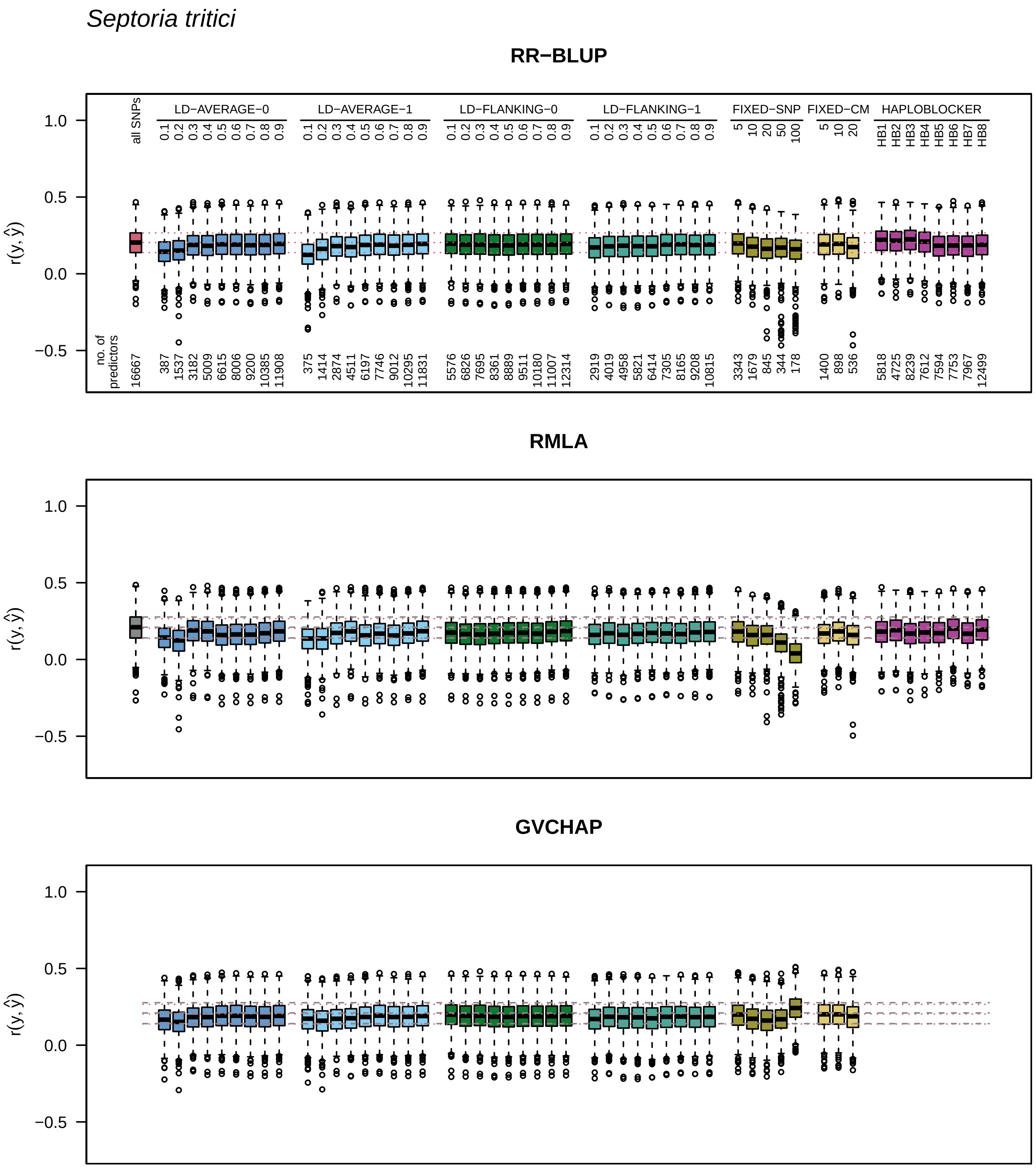

Figure S8. Prediction accuracies for genomic prediction of the resistance score for *Septoria tritici* with different types of haplotype blocks and estimation methods. The boxplots show the correlations $r(y, \hat{y})$ between the observed phenotypic values y and the predicted phenotypic values $\hat{y}$ in the validation set for 1000 cross-validation runs. Haplotype blocks were built based on linkage disequilibrium (`LD-AVERAGE-0`, `LD-AVERAGE-1`, `LD-FLANKING-0`, `LD-FLANKING-1`) with different threshold values $t = 0.1, 0.2, ..., 0.9$ for r^2, with fixed numbers of SNPs per block (`FIXED-SNP`), with a fixed block length in cM (`FIXED-CM`), or with the R package HaploBlocker (`HAPLOBLOCKER`). Red dotted lines: Quartiles from RR-BLUP with 16,667 SNPs (baseline). Gray dashed lines: Quartiles from RMLA with 16,667 SNPs. The number of predictors is the combined number of haplotype blocks and unassigned SNPs.

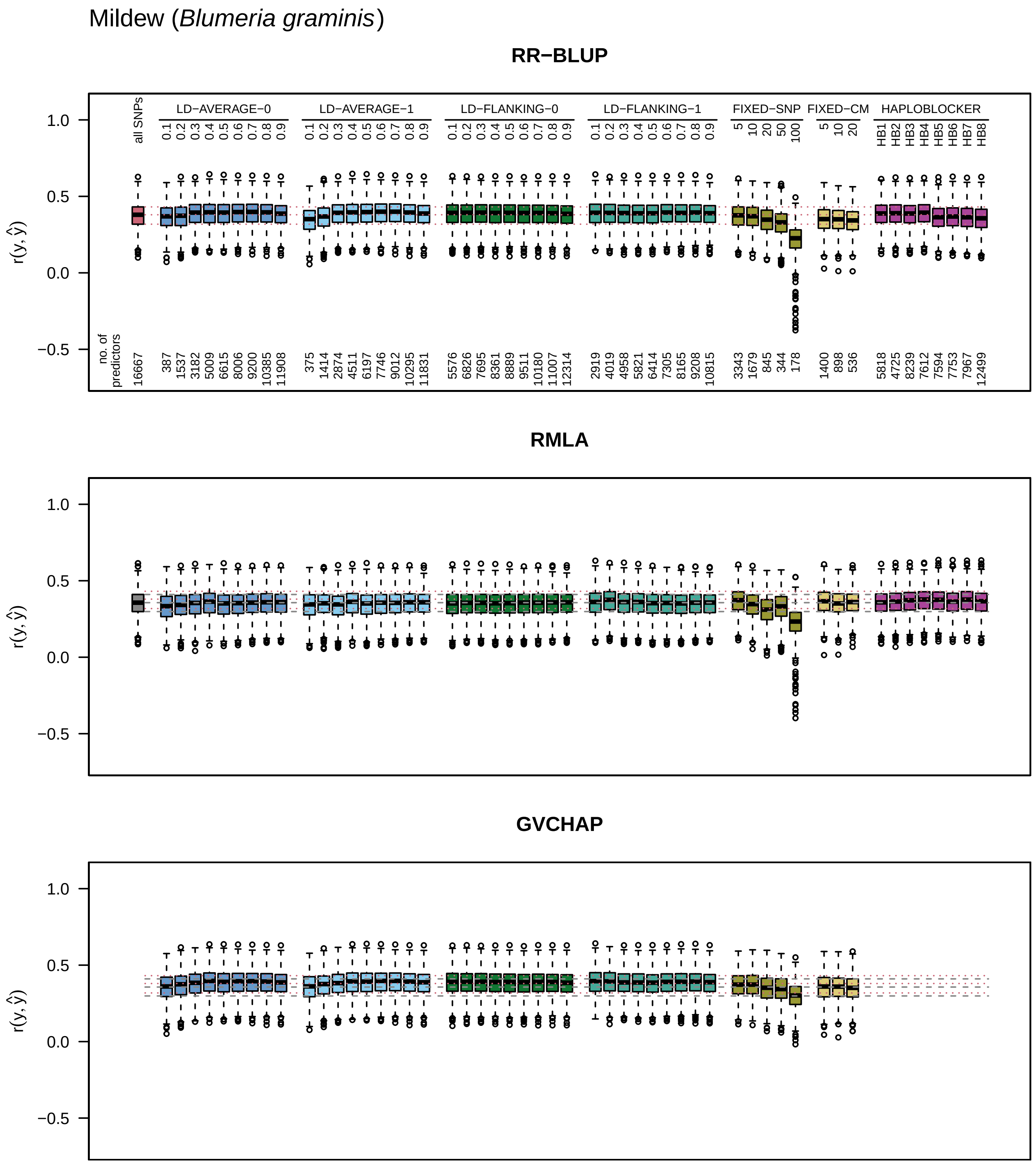

Figure S9. Prediction accuracies for genomic prediction of the resistance score for mildew (*Blumeria graminis*) with different types of haplotype blocks and estimation methods. The boxplots show the correlations $r(y, \hat{y})$ between the observed phenotypic values y and the predicted phenotypic values $\hat{y}$ in the validation set for 1000 cross-validation runs. Haplotype blocks were built based on linkage disequilibrium (`LD-AVERAGE-0`, `LD-AVERAGE-1`, `LD-FLANKING-0`, `LD-FLANKING-1`) with different threshold values $t = 0.1, 0.2, ..., 0.9$ for r^2, with fixed numbers of SNPs per block (`FIXED-SNP`), with a fixed block length in cM (`FIXED-CM`), or with the R package HaploBlocker (`HAPLOBLOCKER`). Red dotted lines: Quartiles from RR-BLUP with 16,667 SNPs (baseline). Gray dashed lines: Quartiles from RMLA with 16,667 SNPs. The number of predictors is the combined number of haplotype blocks and unassigned SNPs.

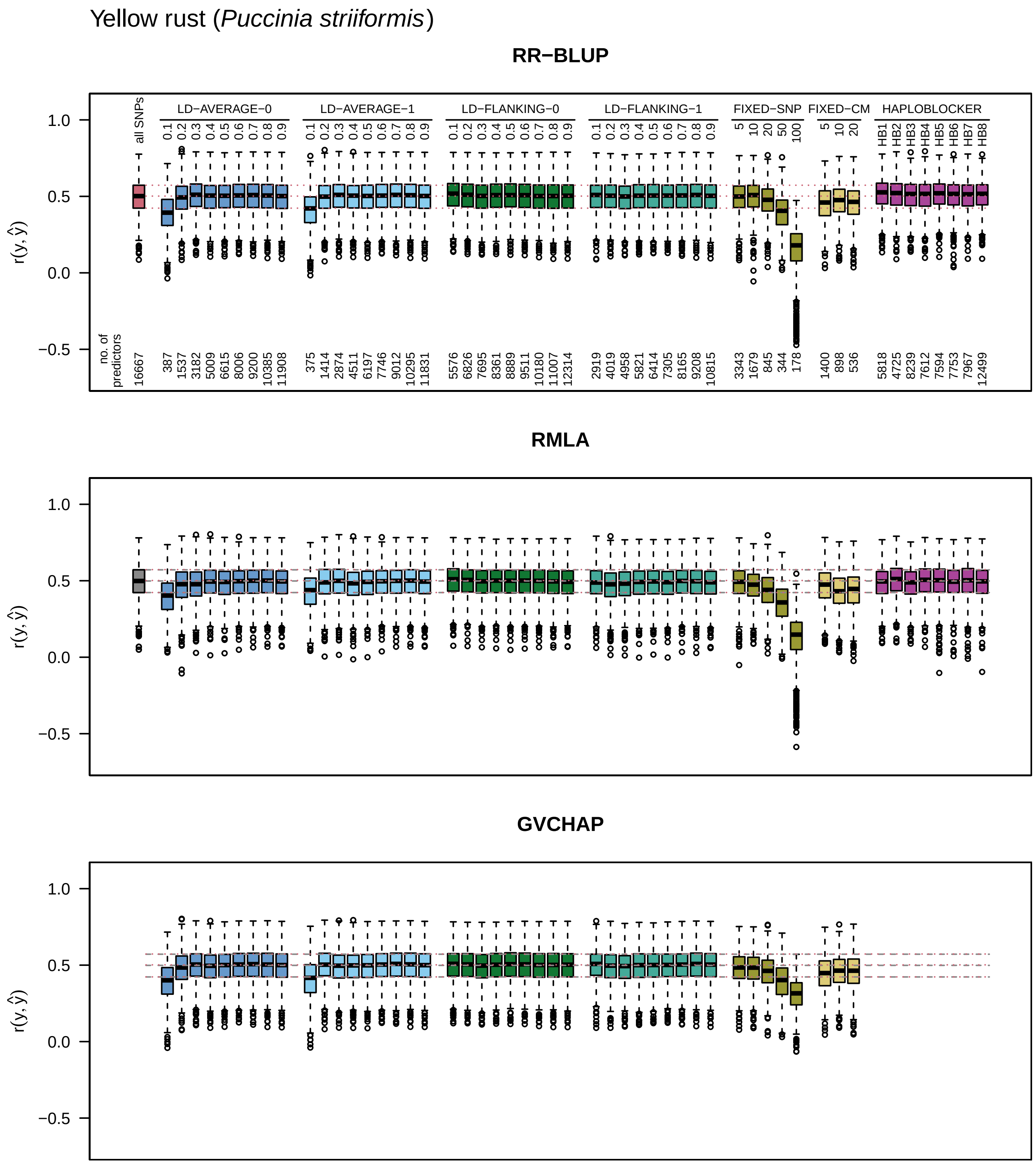

Figure S10. Prediction accuracies for genomic prediction of the resistance score for yellow rust (*Puccinia striiformis*) with different types of haplotype blocks and estimation methods. The boxplots show the correlations $r(y, \hat{y})$ between the observed phenotypic values y and the predicted phenotypic values $\hat{y}$ in the validation set for 1000 cross-validation runs. Haplotype blocks were built based on linkage disequilibrium (`LD-AVERAGE-0`, `LD-AVERAGE-1`, `LD-FLANKING-0`, `LD-FLANKING-1`) with different threshold values $t = 0.1, 0.2, ..., 0.9$ for r^2, with fixed numbers of SNPs per block (`FIXED-SNP`), with a fixed block length in cM (`FIXED-CM`), or with the R package HaploBlocker (`HAPLOBLOCKER`). Red dotted lines: Quartiles from RR-BLUP with 16,667 SNPs (baseline). Gray dashed lines: Quartiles from RMLA with 16,667 SNPs. The number of predictors is the combined number of haplotype blocks and unassigned SNPs.

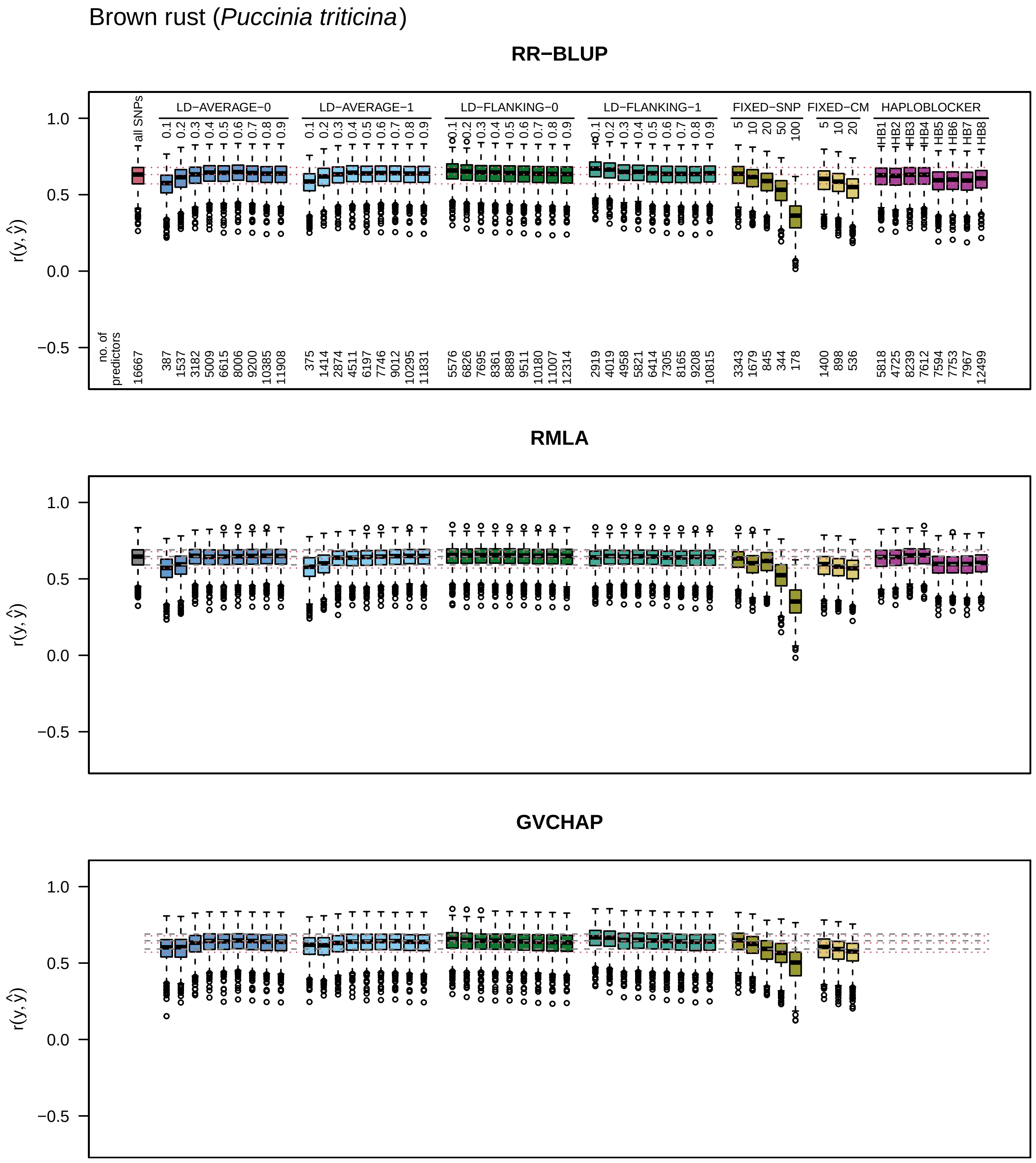

Figure S11. Prediction accuracies for genomic prediction of the resistance score for brown rust (*Puccinia triticina*) with different types of haplotype blocks and estimation methods. The boxplots show the correlations $r(y, \hat{y})$ between the observed phenotypic values y and the predicted phenotypic values $\hat{y}$ in the validation set for 1000 cross-validation runs. Haplotype blocks were built based on linkage disequilibrium (`LD-AVERAGE-0`, `LD-AVERAGE-1`, `LD-FLANKING-0`, `LD-FLANKING-1`) with different threshold values $t = 0.1, 0.2, ..., 0.9$ for r^2, with fixed numbers of SNPs per block (`FIXED-SNP`), with a fixed block length in cM (`FIXED-CM`), or with the R package HaploBlocker (`HAPLOBLOCKER`). Red dotted lines: Quartiles from RR-BLUP with 16,667 SNPs (baseline). Gray dashed lines: Quartiles from RMLA with 16,667 SNPs. The number of predictors is the combined number of haplotype blocks and unassigned SNPs.

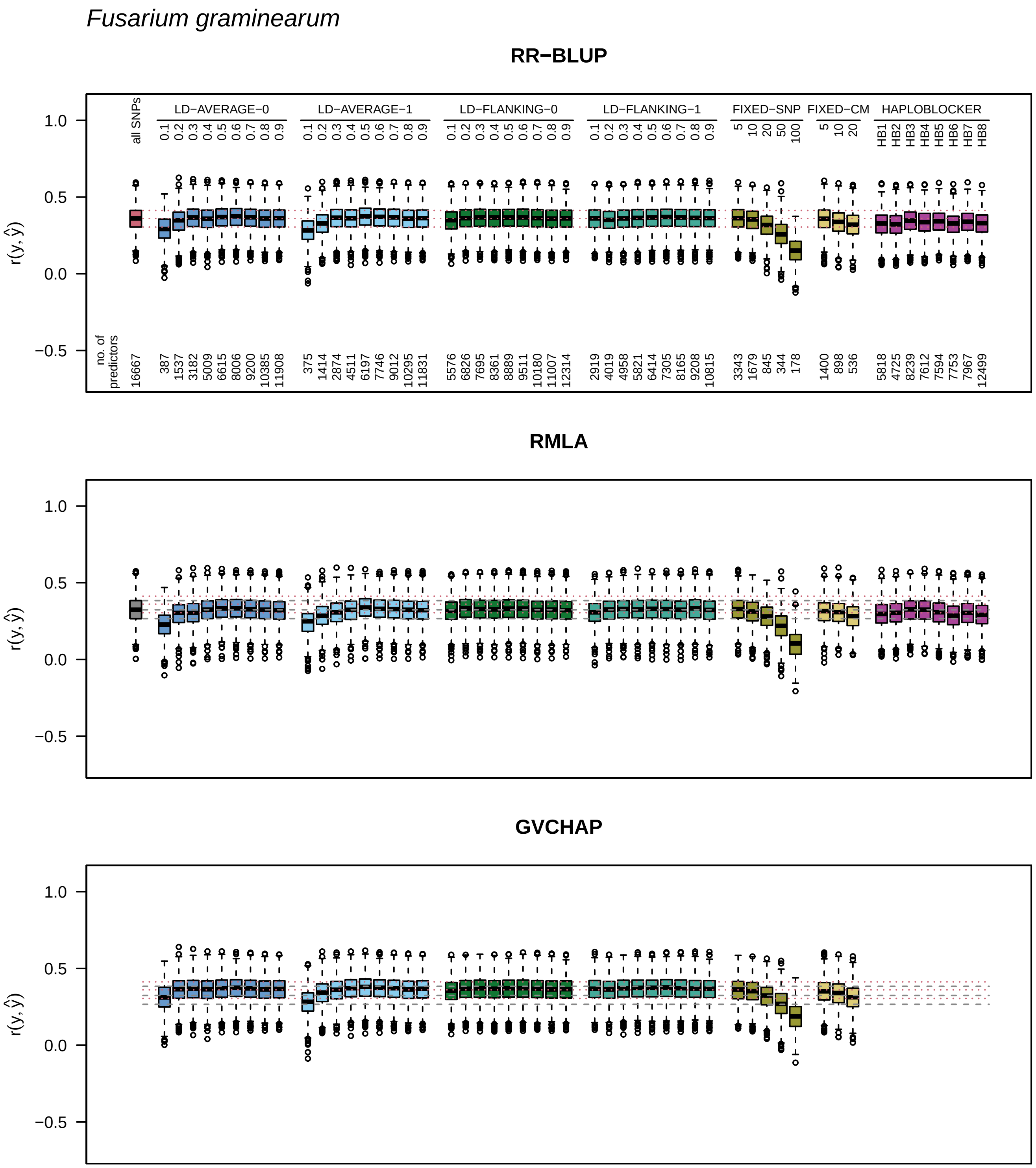

Figure S12. Prediction accuracies for genomic prediction of the resistance score for *Fusarium graminearum* with different types of haplotype blocks and estimation methods. The boxplots show the correlations $r(y, \hat{y})$ between the observed phenotypic values y and the predicted phenotypic values $\hat{y}$ in the validation set for 1000 cross-validation runs. Haplotype blocks were built based on linkage disequilibrium (LD-AVERAGE-0, LD-AVERAGE-1, LD-FLANKING-0, LD-FLANKING-1) with different threshold values $t = 0.1, 0.2, ..., 0.9$ for r^2, with fixed numbers of SNPs per block (FIXED-SNP), with a fixed block length in cM (FIXED-CM), or with the R package HaploBlocker (HAPLOBLOCKER). Red dotted lines: Quartiles from RR-BLUP with 16,667 SNPs (baseline). Gray dashed lines: Quartiles from RMLA with 16,667 SNPs. The number of predictors is the combined number of haplotype blocks and unassigned SNPs.
