# Supplementary material for: Genomic prediction with haplotype blocks in wheat
Source: Front Plant Sci. 2023 May 9;14:1168547. doi: 10.3389/fpls.2023.1168547 (PMC10203549; doi:10.3389/fpls.2023.1168547)
Supplement: Supplementary file 2 [file DataSheet_2.pdf]

frontiers

Supplementary Material

1 MARKER DATA

The following example illustrates (1) how haplotype blocks are built from marker data and (2) how re-parameterized design matrices are constructed from the haplotype blocks.

The example data is for 10 genotypes ($G01, \ldots, G10$) and 10 consecutive SNP markers ($m01, \ldots, m10$) on a single chromosome. The original matrix with phased SNP data (A: 1, C: 2; G: 3; T: 4; missing: -1) is

$$
\begin{array}{c|cccccccccc}
 & m01 & m02 & m03 & m04 & m05 & m06 & m07 & m08 & m09 & m10 \\
\hline
G01 & 4/4 & 1/1 & 3/3 & 1/1 & 4/4 & 2/2 & 4/4 & 2/2 & 3/3 & 3/3 \\
G02 & 2/4 & -1/-1 & 3/3 & 1/1 & 3/3 & 2/4 & 4/4 & 2/2 & 3/3 & 3/3 \\
G03 & 2/2 & 1/3 & 3/3 & 1/1 & 3/3 & 4/4 & 4/4 & 2/2 & 3/3 & 3/3 \\
G04 & 2/2 & 1/1 & 3/3 & 1/1 & 4/4 & 2/2 & 2/2 & 4/4 & 2/2 & 4/4 \\
G05 & 2/2 & 1/3 & 1/1 & 2/2 & 4/4 & 4/4 & 4/4 & 2/2 & 3/3 & 3/3 \\
G06 & 2/2 & 3/3 & 1/1 & 2/2 & 3/3 & 4/4 & 2/4 & 2/2 & 2/2 & 3/3 \\
G07 & 2/2 & 1/1 & 3/3 & 1/1 & 4/4 & 2/2 & 2/2 & 4/4 & 2/2 & 4/4 \\
G08 & 4/4 & 3/3 & 1/1 & 2/2 & 3/3 & 4/4 & 4/4 & 2/2 & 3/3 & 3/3 \\
G09 & 4/4 & 1/3 & 1/1 & 1/1 & 4/4 & 4/4 & 2/2 & 4/4 & 2/2 & 4/4 \\
G10 & 2/4 & 3/3 & 1/1 & 2/2 & 3/3 & 4/4 & 2/2 & 2/4 & 2/2 & 4/4
\end{array}
$$

which is recoded to the design matrix $\mathbf{Z}$ of a marker model

$$
\begin{array}{c|cccccccccc}
 & m01 & m02 & m03 & m04 & m05 & m06 & m07 & m08 & m09 & m10 \\
\hline
G01 & 2 & 0 & 2 & 0 & 2 & 0 & 2 & 0 & 2 & 0 \\
G02 & 1 & \mathrm{NA} & 2 & 0 & 0 & 1 & 2 & 0 & 2 & 0 \\
G03 & 0 & 1 & 2 & 0 & 0 & 2 & 2 & 0 & 2 & 0 \\
G04 & 0 & 0 & 2 & 0 & 2 & 0 & 0 & 2 & 0 & 2 \\
G05 & 0 & 1 & 0 & 2 & 2 & 2 & 2 & 0 & 2 & 0 \\
G06 & 0 & 2 & 0 & 2 & 0 & 2 & 1 & 0 & 0 & 0 \\
G07 & 0 & 0 & 2 & 0 & 2 & 0 & 0 & 2 & 0 & 2 \\
G08 & 2 & 2 & 0 & 2 & 0 & 2 & 2 & 0 & 2 & 0 \\
G09 & 2 & 1 & 0 & 0 & 2 & 2 & 0 & 2 & 0 & 2 \\
G10 & 1 & 2 & 0 & 2 & 0 & 2 & 0 & 1 & 0 & 2
\end{array}
$$

$$
\text{where } X = \begin{cases} 0 & \text{if the genotype is homozygous for the major allele at the SNP} \\ 1 & \text{if the genotype is heterozygous at the SNP} \\ 2 & \text{if the genotype is homozygous for the minor allele at the SNP} \end{cases}
$$

2 LD-BASED HAPLOTYPE BLOCKS

r^2 as a measure for pairwise LD is calculated between all the marker pairs, resulting in the following matrix (r^2 values in the upper diagonal):

	$m01$	$m02$	$m03$	$m04$	$m05$	$m06$	$m07$	$m08$	$m09$	$m10$
$m01$	1.00	0.01	0.04	0.00	0.00	0.03	0.02	0.00	0.04	0.00
$m02$	0.00	1.00	0.34	0.35	0.34	0.36	0.02	0.04	0.01	0.04
$m03$	0.00	0.00	1.00	0.67	0.04	0.54	0.01	0.00	0.04	0.00
$m04$	0.00	0.00	0.00	1.00	0.17	0.36	0.02	0.06	0.00	0.06
$m05$	0.00	0.00	0.00	0.00	1.00	0.27	0.09	0.17	0.04	0.17
$m06$	0.00	0.00	0.00	0.00	0.00	1.00	0.03	0.07	0.01	0.07
$m07$	0.00	0.00	0.00	0.00	0.00	0.00	1.00	0.81	0.82	0.81
$m08$	0.00	0.00	0.00	0.00	0.00	0.00	0.00	1.00	0.67	1.00
$m09$	0.00	0.00	0.00	0.00	0.00	0.00	0.00	0.00	1.00	0.67
$m10$	0.00	0.00	0.00	0.00	0.00	0.00	0.00	0.00	0.00	1.00

Haplotype blocks are built with method `LD-FLANKING-0` with a threshold value $t = 0.3$ for r^2. The following steps are applied:

1. *Search for the pair of adjacent loci on the chromosome that has the greatest LD value r^2 among all pairs of loci that are not yet assigned to a haplotype block.*

	$m01$	$m02$	$m03$	$m04$	$m05$	$m06$	$m07$	$m08$	$m09$	$m10$
$m01$	1.00	0.01	0.04	0.00	0.00	0.03	0.02	0.00	0.04	0.00
$m02$	0.00	1.00	0.34	0.35	0.34	0.36	0.02	0.09	0.01	0.04
$m03$	0.00	0.00	1.00	0.67	0.04	0.54	0.01	0.01	0.04	0.00
$m04$	0.00	0.00	0.00	1.00	0.17	0.36	0.02	0.15	0.00	0.06
$m05$	0.00	0.00	0.00	0.00	1.00	0.27	0.09	0.27	0.04	0.17
$m06$	0.00	0.00	0.00	0.00	0.00	1.00	0.03	0.12	0.01	0.07
$m07$	0.00	0.00	0.00	0.00	0.00	0.00	1.00	0.66	0.82	0.81
$m08$	0.00	0.00	0.00	0.00	0.00	0.00	0.00	1.00	0.54	0.81
$m09$	0.00	0.00	0.00	0.00	0.00	0.00	0.00	0.00	1.00	0.67
$m10$	0.00	0.00	0.00	0.00	0.00	0.00	0.00	0.00	0.00	1.00

The r^2 value for markers $m09$ and $m10$ is 0.67. Note that r^2 between markers $m07$ and $m09$ is higher (0.82) but those markers are not adjacent. Neither are the markers $m07$ and $m09$, or $m07$ and $m10$.

2. *Check whether r^2 is greater than the defined threshold t.*
0.67 > 0.3. It follows that markers $m09$ and $m10$ are grouped into a block.

3. *Check whether the block can be extendend to the left or to the right by comparing t with the LD between the new marker and the marker flanking the block. If $r^2 > t$, extend the block. If $r^2 < t$, then each locus on the chromosome is an individual block.*

	$m01$	$m02$	$m03$	$m04$	$m05$	$m06$	$m07$	$m08$	$m09$	$m10$
$m01$	1.00	0.01	0.04	0.00	0.00	0.03	0.02	0.00	0.04	0.00
$m02$	0.00	1.00	0.34	0.35	0.34	0.36	0.02	0.09	0.01	0.04
$m03$	0.00	0.00	1.00	0.67	0.04	0.54	0.01	0.01	0.04	0.00
$m04$	0.00	0.00	0.00	1.00	0.17	0.36	0.02	0.15	0.00	0.06
$m05$	0.00	0.00	0.00	0.00	1.00	0.27	0.09	0.27	0.04	0.17
$m06$	0.00	0.00	0.00	0.00	0.00	1.00	0.03	0.12	0.01	0.07
$m07$	0.00	0.00	0.00	0.00	0.00	0.00	1.00	0.66	0.82	0.81
$m08$	0.00	0.00	0.00	0.00	0.00	0.00	0.00	1.00	0.54	0.81
$m09$	0.00	0.00	0.00	0.00	0.00	0.00	0.00	0.00	1.00	0.67
$m10$	0.00	0.00	0.00	0.00	0.00	0.00	0.00	0.00	0.00	1.00

The new block is indicated by a vertical line in the column names. To the left, t has to be compared with an LD value of $r^2 = 0.81$ between markers $m10$ (the marker flanking the new block on the right) and $m08$ (the marker flanking the new block on the left). Since $0.81 > 0.3$, the block is extended to the left. Therefore, markers $m08$, $m09$, and $m10$ are grouped into one haplotype block. The block cannot be extended to the right because the chromosome ends with marker $m10$.

4. *Repeat the previous steps until all loci on a chromosome are assigned to a block.*

	$m01$	$m02$	$m03$	$m04$	$m05$	$m06$	$m07$	$m08$	$m09$	$m10$
$m01$	1.00	0.01	0.04	0.00	0.00	0.03	0.02	0.00	0.04	0.00
$m02$	0.00	1.00	0.34	0.35	0.34	0.36	0.02	0.09	0.01	0.04
$m03$	0.00	0.00	1.00	0.67	0.04	0.54	0.01	0.01	0.04	0.00
$m04$	0.00	0.00	0.00	1.00	0.17	0.36	0.02	0.15	0.00	0.06
$m05$	0.00	0.00	0.00	0.00	1.00	0.27	0.09	0.27	0.04	0.17
$m06$	0.00	0.00	0.00	0.00	0.00	1.00	0.03	0.12	0.01	0.07
$m07$	0.00	0.00	0.00	0.00	0.00	0.00	1.00	0.66	0.82	0.81
$m08$	0.00	0.00	0.00	0.00	0.00	0.00	0.00	1.00	0.54	0.81
$m09$	0.00	0.00	0.00	0.00	0.00	0.00	0.00	0.00	1.00	0.67
$m10$	0.00	0.00	0.00	0.00	0.00	0.00	0.00	0.00	0.00	1.00

$0.81 > 0.3$. The block can be extended to the left and marker $m07$ is included.

$$
\begin{array}{c|cccccc|cccc}
 & m01 & m02 & m03 & m04 & m05 & m06 & m07 & m08 & m09 & m10 \\
\hline
m01 & 1.00 & 0.01 & 0.04 & 0.00 & 0.00 & 0.03 & 0.02 & 0.00 & 0.04 & 0.00 \\
m02 & 0.00 & 1.00 & 0.34 & 0.35 & 0.34 & 0.36 & 0.02 & 0.09 & 0.01 & 0.04 \\
m03 & 0.00 & 0.00 & 1.00 & 0.67 & 0.04 & 0.54 & 0.01 & 0.01 & 0.04 & 0.00 \\
m04 & 0.00 & 0.00 & 0.00 & 1.00 & 0.17 & 0.36 & 0.02 & 0.15 & 0.00 & 0.06 \\
m05 & 0.00 & 0.00 & 0.00 & 0.00 & 1.00 & 0.27 & 0.09 & 0.27 & 0.04 & 0.17 \\
m06 & 0.00 & 0.00 & 0.00 & 0.00 & 0.00 & 1.00 & 0.03 & 0.12 & 0.01 & \color{red}{0.07} \\
m07 & 0.00 & 0.00 & 0.00 & 0.00 & 0.00 & 0.00 & 1.00 & 0.66 & 0.82 & 0.81 \\
m08 & 0.00 & 0.00 & 0.00 & 0.00 & 0.00 & 0.00 & 0.00 & 1.00 & 0.54 & 0.81 \\
m09 & 0.00 & 0.00 & 0.00 & 0.00 & 0.00 & 0.00 & 0.00 & 0.00 & 1.00 & 0.67 \\
m10 & 0.00 & 0.00 & 0.00 & 0.00 & 0.00 & 0.00 & 0.00 & 0.00 & 0.00 & 1.00
\end{array}
$$

$0.07 < 0.3$. The block cannot be extended to the left. Now search for the pair of adjacent loci on the chromosome that are not yet assigned to a haplotype block.

$$
\begin{array}{c|cccccc|cccc}
 & m01 & m02 & m03 & m04 & m05 & m06 & m07 & m08 & m09 & m10 \\
\hline
m01 & 1.00 & 0.01 & 0.04 & 0.00 & 0.00 & 0.03 & 0.02 & 0.00 & 0.04 & 0.00 \\
m02 & 0.00 & 1.00 & 0.34 & 0.35 & 0.34 & 0.36 & 0.02 & 0.09 & 0.01 & 0.04 \\
m03 & 0.00 & 0.00 & 1.00 & \color{red}{0.67} & 0.04 & 0.54 & 0.01 & 0.01 & 0.04 & 0.00 \\
m04 & 0.00 & 0.00 & 0.00 & 1.00 & 0.17 & 0.36 & 0.02 & 0.15 & 0.00 & 0.06 \\
m05 & 0.00 & 0.00 & 0.00 & 0.00 & 1.00 & 0.27 & 0.09 & 0.27 & 0.04 & 0.17 \\
m06 & 0.00 & 0.00 & 0.00 & 0.00 & 0.00 & 1.00 & 0.03 & 0.12 & 0.01 & 0.07 \\
m07 & 0.00 & 0.00 & 0.00 & 0.00 & 0.00 & 0.00 & 1.00 & 0.66 & 0.82 & 0.81 \\
m08 & 0.00 & 0.00 & 0.00 & 0.00 & 0.00 & 0.00 & 0.00 & 1.00 & 0.54 & 0.81 \\
m09 & 0.00 & 0.00 & 0.00 & 0.00 & 0.00 & 0.00 & 0.00 & 0.00 & 1.00 & 0.67 \\
m10 & 0.00 & 0.00 & 0.00 & 0.00 & 0.00 & 0.00 & 0.00 & 0.00 & 0.00 & 1.00
\end{array}
$$

$0.67 > 0.3$. Markers $m03$ and $m04$ are grouped into a haplotype block.

$$
\begin{array}{c|cc|cc|cc|cccc}
 & m01 & m02 & m03 & m04 & m05 & m06 & m07 & m08 & m09 & m10 \\
\hline
m01 & 1.00 & 0.01 & 0.04 & 0.00 & 0.00 & 0.03 & 0.02 & 0.00 & 0.04 & 0.00 \\
m02 & 0.00 & 1.00 & 0.34 & \color{red}{0.35} & 0.34 & 0.36 & 0.02 & 0.09 & 0.01 & 0.04 \\
m03 & 0.00 & 0.00 & 1.00 & 0.67 & 0.04 & 0.54 & 0.01 & 0.01 & 0.04 & 0.00 \\
m04 & 0.00 & 0.00 & 0.00 & 1.00 & 0.17 & 0.36 & 0.02 & 0.15 & 0.00 & 0.06 \\
m05 & 0.00 & 0.00 & 0.00 & 0.00 & 1.00 & 0.27 & 0.09 & 0.27 & 0.04 & 0.17 \\
m06 & 0.00 & 0.00 & 0.00 & 0.00 & 0.00 & 1.00 & 0.03 & 0.12 & 0.01 & 0.07 \\
m07 & 0.00 & 0.00 & 0.00 & 0.00 & 0.00 & 0.00 & 1.00 & 0.66 & 0.82 & 0.81 \\
m08 & 0.00 & 0.00 & 0.00 & 0.00 & 0.00 & 0.00 & 0.00 & 1.00 & 0.54 & 0.81 \\
m09 & 0.00 & 0.00 & 0.00 & 0.00 & 0.00 & 0.00 & 0.00 & 0.00 & 1.00 & 0.67 \\
m10 & 0.00 & 0.00 & 0.00 & 0.00 & 0.00 & 0.00 & 0.00 & 0.00 & 0.00 & 1.00
\end{array}
$$

$0.35 > 0.3$. The block can be extended to the left and marker $m02$ is included.

	$m01$	$m02$	$m03$	$m04$	$m05$	$m06$	$m07$	$m08$	$m09$	$m10$
$m01$	1.00	0.01	0.04	0.00	0.00	0.03	0.02	0.00	0.04	0.00
$m02$	0.00	1.00	0.34	0.35	0.34	0.36	0.02	0.09	0.01	0.04
$m03$	0.00	0.00	1.00	0.67	0.04	0.54	0.01	0.01	0.04	0.00
$m04$	0.00	0.00	0.00	1.00	0.17	0.36	0.02	0.15	0.00	0.06
$m05$	0.00	0.00	0.00	0.00	1.00	0.27	0.09	0.27	0.04	0.17
$m06$	0.00	0.00	0.00	0.00	0.00	1.00	0.03	0.12	0.01	0.07
$m07$	0.00	0.00	0.00	0.00	0.00	0.00	1.00	0.66	0.82	0.81
$m08$	0.00	0.00	0.00	0.00	0.00	0.00	0.00	1.00	0.54	0.81
$m09$	0.00	0.00	0.00	0.00	0.00	0.00	0.00	0.00	1.00	0.67
$m10$	0.00	0.00	0.00	0.00	0.00	0.00	0.00	0.00	0.00	1.00

$0 < 0.3$. The block cannot be extended to the left.

	$m01$	$m02$	$m03$	$m04$	$m05$	$m06$	$m07$	$m08$	$m09$	$m10$
$m01$	1.00	0.01	0.04	0.00	0.00	0.03	0.02	0.00	0.04	0.00
$m02$	0.00	1.00	0.34	0.35	0.34	0.36	0.02	0.09	0.01	0.04
$m03$	0.00	0.00	1.00	0.67	0.04	0.54	0.01	0.01	0.04	0.00
$m04$	0.00	0.00	0.00	1.00	0.17	0.36	0.02	0.15	0.00	0.06
$m05$	0.00	0.00	0.00	0.00	1.00	0.27	0.09	0.27	0.04	0.17
$m06$	0.00	0.00	0.00	0.00	0.00	1.00	0.03	0.12	0.01	0.07
$m07$	0.00	0.00	0.00	0.00	0.00	0.00	1.00	0.66	0.82	0.81
$m08$	0.00	0.00	0.00	0.00	0.00	0.00	0.00	1.00	0.54	0.81
$m09$	0.00	0.00	0.00	0.00	0.00	0.00	0.00	0.00	1.00	0.67
$m10$	0.00	0.00	0.00	0.00	0.00	0.00	0.00	0.00	0.00	1.00

$0.34 > 0.3$. The block can be extended to the right and marker $m05$ is included.

	$m01$	$m02$	$m03$	$m04$	$m05$	$m06$	$m07$	$m08$	$m09$	$m10$
$m01$	1.00	0.01	0.04	0.00	0.00	0.03	0.02	0.00	0.04	0.00
$m02$	0.00	1.00	0.34	0.35	0.34	0.36	0.02	0.09	0.01	0.04
$m03$	0.00	0.00	1.00	0.67	0.04	0.54	0.01	0.01	0.04	0.00
$m04$	0.00	0.00	0.00	1.00	0.17	0.36	0.02	0.15	0.00	0.06
$m05$	0.00	0.00	0.00	0.00	1.00	0.27	0.09	0.27	0.04	0.17
$m06$	0.00	0.00	0.00	0.00	0.00	1.00	0.03	0.12	0.01	0.07
$m07$	0.00	0.00	0.00	0.00	0.00	0.00	1.00	0.66	0.82	0.81
$m08$	0.00	0.00	0.00	0.00	0.00	0.00	0.00	1.00	0.54	0.81
$m09$	0.00	0.00	0.00	0.00	0.00	0.00	0.00	0.00	1.00	0.67
$m10$	0.00	0.00	0.00	0.00	0.00	0.00	0.00	0.00	0.00	1.00

$0.36 > 0.3$. The block can be extended to the right and marker $m06$ is included. The haplotype block ends here because a new one begins with marker $m07$. On the left of the haplotype block, only marker $m01$ is left. This marker is converted in a haplotype block with just one marker.

The final assignment of the markers to the haplotype blocks:

hb1: $m01$
hb2: $m02$, $m03$, $m04$, $m05$, $m06$
hb3: $m07$, $m08$, $m09$, $m10$

The corresponding SNPs are

	**hb1**	**hb2**					**hb3**			
	$m01$	$m02$	$m03$	$m04$	$m05$	$m06$	$m07$	$m08$	$m09$	$m10$
$G01$	4/4	1/1	3/3	1/1	4/4	2/2	4/4	2/2	3/3	3/3
$G02$	2/4	−1/−1	3/3	1/1	3/3	2/4	4/4	2/2	3/3	3/3
$G03$	2/2	1/3	3/3	1/1	3/3	4/4	4/4	2/2	3/3	3/3
$G04$	2/2	1/1	3/3	1/1	4/4	2/2	2/2	4/4	2/2	4/4
$G05$	2/2	1/3	1/1	2/2	4/4	4/4	4/4	2/2	3/3	3/3
$G06$	2/2	3/3	1/1	2/2	3/3	4/4	2/4	2/2	2/2	3/3
$G07$	2/2	1/1	3/3	1/1	4/4	2/2	2/2	4/4	2/2	4/4
$G08$	4/4	3/3	1/1	2/2	3/3	4/4	4/4	2/2	3/3	3/3
$G09$	4/4	1/3	1/1	1/1	4/4	4/4	2/2	4/4	2/2	4/4
$G10$	2/4	3/3	1/1	2/2	3/3	4/4	2/4	2/4	2/2	4/4

For $m01$ which forms its own haplotype block $hb1$, there are only two variants, 2 and 4. However, for $hb2$ and $hb3$, more combinations of the original SNP alleles have to be considered. The following matrix shows the possible combinations of marker alleles for $hb2$. There is only one line for each homozygous genotype and two lines for the genotypes that were heterozygous at one of the markers.

	**hb2**				
	$m02$	$m03$	$m04$	$m05$	$m06$
$G01$	1	3	1	4	2
$G02$	−1	3	1	3	2
	−1	3	1	3	4
$G03$	1	3	1	3	4
	3	3	1	3	4
$G04$	1	3	1	4	2
$G05$	1	1	2	4	4
	3	1	2	4	4
$G06$	3	1	2	3	4
$G07$	1	3	1	4	2
$G08$	3	1	2	3	4
$G09$	1	1	1	4	4
	3	1	1	4	4
$G10$	3	1	2	3	4

When combinations that occur more than once are removed, the following sequences of marker alleles are left for $hb2$:

$$
\begin{array}{c}
\boldsymbol{hb2} \\
\begin{array}{ccccc} m02 & m03 & m04 & m05 & m06 \end{array} \\
\begin{pmatrix}
1 & 3 & 1 & 4 & 2 \\
-1 & 3 & 1 & 3 & 2 \\
-1 & 3 & 1 & 3 & 4 \\
1 & 3 & 1 & 3 & 4 \\
3 & 3 & 1 & 3 & 4 \\
1 & 1 & 2 & 4 & 4 \\
3 & 1 & 2 & 4 & 4 \\
3 & 1 & 2 & 3 & 4 \\
1 & 1 & 1 & 4 & 4 \\
3 & 1 & 1 & 4 & 4
\end{pmatrix}
\end{array}
$$

Each of these sequences can be considered a unique variant (or "allele") of haplotype block $hb2$. Note that (1) SNP data must be phased to define meaningful variants and (2) missing marker data are considered as separate alleles.

For haplotype block $hb03$, the derivation of the variants looks as follows:

$$
\boldsymbol{hb3}
$$

$$
\begin{array}{c|cccc}
 & m07 & m08 & m09 & m10 \\
\hline
G01 & 4 & 2 & 3 & 3 \\
G02 & 4 & 2 & 3 & 3 \\
G03 & 4 & 2 & 3 & 3 \\
G04 & 2 & 4 & 2 & 4 \\
G05 & 4 & 2 & 3 & 3 \\
G06 & 2 & 2 & 2 & 3 \\
 & 4 & 2 & 2 & 3 \\
G07 & 2 & 4 & 2 & 4 \\
G08 & 4 & 2 & 3 & 3 \\
G09 & 2 & 4 & 2 & 4 \\
G10 & 2 & 2 & 2 & 4 \\
 & 2 & 4 & 2 & 4
\end{array}
$$

The following unique combinations remain:

$$
\begin{array}{c}
\boldsymbol{hb3} \\
\begin{array}{cccc} m07 & m08 & m09 & m10 \end{array} \\
\begin{pmatrix}
4 & 2 & 3 & 3 \\
2 & 4 & 2 & 4 \\
2 & 2 & 2 & 3 \\
4 & 2 & 2 & 3 \\
2 & 4 & 2 & 4
\end{pmatrix}
\end{array}
$$

Consequently, there are two variants for haplotype block $hb1$, ten variants for haplotype block $hb2$, and five variants for haplotype block $hb3$.

Overview over distinct variants for haplotype blocks built with method `LD-FLANKING-0` for an r^2 threshold of 0.3:

Haplotype block	Variant	Sequence of SNP alleles
$hb1$	1	2
$hb1$	2	4
$hb2$	1	1;3;1;4;2
$hb2$	2	3;1;2;3;4
$hb2$	3	1;3;1;3;4
$hb2$	4	3;3;1;3;4
$hb2$	5	1;1;2;4;4
$hb2$	6	3;1;2;4;4
$hb2$	7	-1;3;1;3;4
$hb2$	8	-1;3;1;3;2
$hb2$	9	1;1;1;4;4
$hb2$	10	3;1;1;4;4
$hb3$	1	4;2;3;3
$hb3$	2	2;4;2;4
$hb3$	3	4;2;2;3
$hb3$	4	2;2;2;4
$hb3$	5	2;2;2;3

The following matrix assigns the haplotype blocks variants to the genotypes according to the SNP sequence that they show for the respective block.

$$\begin{array}{c} \\ G01 \\ G02 \\ G03 \\ G04 \\ G05 \\ G06 \\ G07 \\ G08 \\ G09 \\ G10 \end{array} \begin{array}{c} \begin{array}{ccc} hb1 & hb2 & hb3 \end{array} \\ \begin{pmatrix} 2/2 & 1/1 & 1/1 \\ 1/2 & -1/-1 & 1/1 \\ 1/1 & 3/4 & 1/1 \\ 1/1 & 1/1 & 2/2 \\ 1/1 & 5/6 & 1/1 \\ 1/1 & 2/2 & 5/3 \\ 1/1 & 1/1 & 2/2 \\ 2/2 & 2/2 & 1/1 \\ 2/2 & 9/10 & 2/2 \\ 1/2 & 2/2 & 4/2 \end{pmatrix} \end{array}$$

Compare this to the sequences of SNP alleles of the genotypes in haplotype block *hb*3:

	***hb*3**			
	$m07$	$m08$	$m09$	$m10$
$G01$	4	2	3	3
$G02$	4	2	3	3
$G03$	4	2	3	3
$G04$	2	4	2	4
$G05$	4	2	3	3
$G06$	2	2	2	3
	4	2	2	3
$G07$	2	4	2	4
$G08$	4	2	3	3
$G09$	2	4	2	4
$G10$	2	2	2	4
	2	4	2	4

The matrix with the haplotype variants then has to be re-parametrized in order to obtain a design matrix **Z** with encoding 0,1,2 for the mixed linear model. In this matrix, each variant for each haplotype block gets one column. The variants are then encoded with 0 (haploblock variant is absent), 1 (one copy of the haploblock variant present), or 2 (two copies of the haploblock variant present).

	***hb*1**		***hb*2**								***hb*3**				
	$v01$	$v02$	$v01$	$v02$	$v03$	$v04$	$v05$	$v06$	$v09$	$v10$	$v01$	$v02$	$v03$	$v04$	$v05$
$G01$	0	2	2	0	0	0	0	0	0	0	2	0	0	0	0
$G02$	1	1	0	0	0	0	0	0	0	0	2	0	0	0	0
$G03$	2	0	0	0	1	1	0	0	0	0	2	0	0	0	0
$G04$	2	0	2	0	0	0	0	0	0	0	0	2	0	0	0
$G05$	2	0	0	0	0	0	1	1	0	0	2	0	0	0	0
$G06$	2	0	0	2	0	0	0	0	0	0	0	0	1	0	1
$G07$	2	0	2	0	0	0	0	0	0	0	0	2	0	0	0
$G08$	0	2	0	2	0	0	0	0	0	0	2	0	0	0	0
$G09$	0	2	0	0	0	0	0	0	1	1	0	2	0	0	0
$G10$	1	1	0	2	0	0	0	0	0	0	0	1	0	1	0

This is the R code that can be used to obtain the matrices:

```
> library("SelectionTools")
> # Package can be downloaded from http://population-genetics.uni-giessen.de/˜software/
>
> # Make the marker data file (assumption: phased marker data)
> marker <- data.frame(matrix(c("4/4", "2/4", "2/2", "2/2", "2/2", "2/2", "2/2", "4/4", "4/4", "2/4",
+                               "1/1", "-1/-1", "1/3", "1/1", "1/3", "3/3", "1/1", "3/3", "1/3", "3/3",
+                               "3/3", "3/3", "3/3", "3/3", "1/1", "1/1", "3/3", "1/1", "1/1", "1/1",
+                               "1/1", "1/1", "1/1", "1/1", "2/2", "2/2", "1/1", "2/2", "1/1", "2/2",
+                               "4/4", "3/3", "3/3", "4/4", "4/4", "3/3", "4/4", "3/3", "4/4", "3/3",
```

```
+                                "2/2", "2/4", "4/4", "2/2", "4/4", "4/4", "2/2", "4/4", "4/4", "4/4",
+                                "4/4", "4/4", "4/4", "2/2", "4/4", "2/4", "2/2", "4/4", "2/2", "2/2",
+                                "2/2", "2/2", "2/2", "4/4", "2/2", "2/2", "4/4", "2/2", "4/4", "2/4",
+                                "3/3", "3/3", "3/3", "2/2", "3/3", "2/2", "2/2", "3/3", "2/2", "2/2",
+                                "3/3", "3/3", "3/3", "4/4", "3/3", "3/3", "4/4", "3/3", "4/4", "4/4"),
+                              byrow=T, ncol=10))
> colnames(marker) <- sprintf("G%02i", 1:10)
> rownames(marker) <- sprintf("m%02i", 1:10)
> write.table(marker, "example-marker.txt", quote=F)
>
> # Make the map file
> map <- data.frame(name = sprintf("m%02i", 1:10),
+                   chrom = 1,
+                   pos = seq(from=1, to=100, by=10))
> write.table(map, "example-map.txt", quote=F, row.names=F, sep=" ")
>
> # Read marker data and map file
> st.read.marker.data("example-marker.txt", format="m", data.set="default")
M (data set 'default'): No. of individuals: 10, no. of markers: 10
> st.read.map("example-map.txt", format="mcp", skip=1, data.set="default")
M (data set 'default'): No. of individuals: 10, no. of markers: 10
>
> # Marker data in SelectionTools
> xx <- st.marker.data.statistics()
M (data set 'default'): No. of individuals: 10, no. of markers: 10
> xx$genotypes # note the swap of rows and columns!
   Mar/Ind G01   G02 G03 G04 G05 G06 G07 G08 G09 G10
1      m01 4/4   2/4 2/2 2/2 2/2 2/2 2/2 4/4 4/4 2/4
2      m02 1/1 -1/-1 1/3 1/1 1/3 3/3 1/1 3/3 1/3 3/3
3      m03 3/3   3/3 3/3 3/3 1/1 1/1 3/3 1/1 1/1 1/1
4      m04 1/1   1/1 1/1 1/1 2/2 2/2 1/1 2/2 1/1 2/2
5      m05 4/4   3/3 3/3 4/4 4/4 3/3 4/4 3/3 4/4 3/3
6      m06 2/2   2/4 4/4 2/2 4/4 4/4 2/2 4/4 4/4 4/4
7      m07 4/4   4/4 4/4 2/2 4/4 2/4 2/2 4/4 2/2 2/2
8      m08 2/2   2/2 2/2 4/4 2/2 2/2 4/4 2/2 4/4 2/4
9      m09 3/3   3/3 3/3 2/2 3/3 2/2 2/2 3/3 2/2 2/2
10     m10 3/3   3/3 3/3 4/4 3/3 3/3 4/4 3/3 4/4 4/4
>
> # Design matrix with single markers
> ZZ <- gs.build.Z(data.set="default", out.filename="Z.matrix", auxfiles=T)
> ZZ
    m01.4 m02.3 m03.3 m04.2 m05.4 m06.4 m07.4 m08.4 m09.3 m10.4
G01     2     0     2     0     2     0     2     0     2     0
G02     1     1     2     0     0     1     2     0     2     0
G03     0     1     2     0     0     2     2     0     2     0
G04     0     0     2     0     2     0     0     2     0     2
G05     0     1     0     2     2     2     2     0     2     0
G06     0     2     0     2     0     2     1     0     0     0
G07     0     0     2     0     2     0     0     2     0     2
```

```
G08     2     2     0     2     0     2     2     0     2     0
G09     2     1     0     0     2     2     0     2     0     2
G10     1     2     0     2     0     2     0     1     0     2
>
> # Calculate LD
> ld <- st.calc.ld ( ld.measure="r2",
+                    data.set="default" )
> head(ld, 10)
   Chrom Locus1 Locus2 Name1 Name2       LD
1      1      1      2   m01   m02 0.009848
2      1      1      3   m01   m03 0.041667
3      1      1      4   m01   m04 0.001736
4      1      1      5   m01   m05 0.000000
5      1      1      6   m01   m06 0.029304
6      1      1      7   m01   m07 0.015152
7      1      1      8   m01   m08 0.001832
8      1      1      9   m01   m09 0.041667
9      1      1     10   m01   m10 0.001736
10     1      2      3   m02   m03 0.336364
>
> # Pairwise LD
> pairwise.ld <- xtabs(LD ~ Name1 + Name2, data=ld)
> round(pairwise.ld, 2)
     Name2
Name1  m02  m03  m04  m05  m06  m07  m08  m09  m10
  m01 0.01 0.04 0.00 0.00 0.03 0.02 0.00 0.04 0.00
  m02 0.00 0.34 0.35 0.34 0.36 0.02 0.09 0.01 0.04
  m03 0.00 0.00 0.67 0.04 0.54 0.01 0.01 0.04 0.00
  m04 0.00 0.00 0.00 0.17 0.36 0.02 0.15 0.00 0.06
  m05 0.00 0.00 0.00 0.00 0.27 0.09 0.27 0.04 0.17
  m06 0.00 0.00 0.00 0.00 0.00 0.03 0.12 0.01 0.07
  m07 0.00 0.00 0.00 0.00 0.00 0.00 0.66 0.82 0.81
  m08 0.00 0.00 0.00 0.00 0.00 0.00 0.00 0.54 0.81
  m09 0.00 0.00 0.00 0.00 0.00 0.00 0.00 0.00 0.67
>
> # Define haplotype blocks
> hb <- st.def.hblocks ( ld.threshold = 0.3, # Minimum LD
+                        ld.criterion = "flanking", # between markers
+                        data.set="default" ) # flanking the block
M (data set 'default'): LD of markers flanking haplotype blocks > 0.30
> hb
  Chrom Pos    Name Class              Markers
1     1   1 b000000     b                 m01;
2     1  31 b000001     b m02;m03;m04;m05;m06;
3     1  76 b000002     b     m07;m08;m09;m10;
>
> # Recode data set
> rb <- st.recode.hil(data.set="default")
M (data set 'default'): No. of individuals: 10, no. of markers: 3
```

```
> rb
     Block AlleleNr  AlleleDef
1  b000000        1          2
2  b000000        2          4
3  b000001        1  1;3;1;4;2
4  b000001        2  3;1;2;3;4
5  b000001        3  1;3;1;3;4
6  b000001        4  3;3;1;3;4
7  b000001        5  1;1;2;4;4
8  b000001        6  3;1;2;4;4
9  b000001        7 -1;3;1;3;4
10 b000001        8 -1;3;1;3;2
11 b000001        9  1;1;1;4;4
12 b000001       10  3;1;1;4;4
13 b000002        1    4;2;3;3
14 b000002        2    2;4;2;4
15 b000002        3    4;2;2;3
16 b000002        4    2;2;2;4
17 b000002        5    2;2;2;3
>
> # Variants for haplotype blocks
> xx <- st.marker.data.statistics("default")
M (data set 'default'): No. of individuals: 10, no. of markers: 3
> xx
$individual.list
   Name    InMis
1   G01 0.000000
2   G02 0.333333
3   G03 0.000000
4   G04 0.000000
5   G05 0.000000
6   G06 0.000000
7   G07 0.000000
8   G08 0.000000
9   G09 0.000000
10  G10 0.000000

$marker.list
     Name NoAll MaMis ExHet AM A1 A2 A3 A4 A5 A6 A9 A10
1 b000000     2   0.0 0.480  0 12  8  0  0  0  0  0   0
2 b000001     8   0.1 0.759  2  6  6  1  1  1  1  1   1
3 b000002     5   0.0 0.620  0 10  7  1  1  1  0  0   0

$genotypes
  Mar/Ind G01   G02 G03 G04 G05 G06 G07 G08  G09 G10
1 b000000 2/2   1/2 1/1 1/1 1/1 1/1 1/1 2/2  2/2 1/2
2 b000001 1/1 -1/-1 3/4 1/1 5/6 2/2 1/1 2/2 9/10 2/2
3 b000002 1/1   1/1 1/1 2/2 1/1 5/3 2/2 1/1  2/2 4/2
```

```
>
> # Write data file and read it in again - this way, one column is created
> # for each variant of each haplotype block
> st.write.marker.data(nfilename="example-hb-ld", format="n", data.set="default")
> XX <- read.table("example-hb-ld.npo", header=T)
> XX <- data.frame(t(XX))
> rownames(XX) <- sprintf("G%02i", 1:10)
> XX
    b000000.1 b000000.2 b000001.1 b000001.2 b000001.3 b000001.4 b000001.5 b000001.6 b000001.9
G01         0         1         1         0         0         0         0         0         0
G02         1         1         0         0         0         0         0         0         0
G03         1         0         0         0         1         1         0         0         0
G04         1         0         1         0         0         0         0         0         0
G05         1         0         0         0         0         0         1         1         0
G06         1         0         0         1         0         0         0         0         0
G07         1         0         1         0         0         0         0         0         0
G08         0         1         0         1         0         0         0         0         0
G09         0         1         0         0         0         0         0         0         1
G10         1         1         0         1         0         0         0         0         0
    b000001.10 b000002.1 b000002.2 b000002.3 b000002.4 b000002.5
G01          0         1         0         0         0         0
G02          0         1         0         0         0         0
G03          0         1         0         0         0         0
G04          0         0         1         0         0         0
G05          0         1         0         0         0         0
G06          0         0         0         1         0         1
G07          0         0         1         0         0         0
G08          0         1         0         0         0         0
G09          1         0         1         0         0         0
G10          0         0         1         0         1         0
>
> # The matrix must be re-coded so that homo- and heterozygous individuals
> # can be distinguished.
>
> no.alleles <- xx$marker.list$NoAll
> no.hb <- length(no.alleles)
> geno <- xx$individual.list$Name
> counter <- 1
>
> for (ii in 1:no.hb) {
+   alleles <- no.alleles[ii]
+   XX.subset <- XX[,counter:(counter+alleles-1)]
+
+   for (GEN in geno) {
+
+     rowsum <- sum(XX.subset[GEN,])
+
+     if (rowsum == 1) {
+       XX[GEN,counter:(counter+alleles-1)][XX[GEN,counter:(counter+alleles-1)]==1] <- 2
```

```
+     }
+
+   }
+
+   counter <- counter + alleles
+ }
>
> XX
    b000000.1 b000000.2 b000001.1 b000001.2 b000001.3 b000001.4 b000001.5 b000001.6 b000001.9
G01         0         2         2         0         0         0         0         0         0
G02         1         1         0         0         0         0         0         0         0
G03         2         0         0         0         1         1         0         0         0
G04         2         0         2         0         0         0         0         0         0
G05         2         0         0         0         0         0         1         1         0
G06         2         0         0         2         0         0         0         0         0
G07         2         0         2         0         0         0         0         0         0
G08         0         2         0         2         0         0         0         0         0
G09         0         2         0         0         0         0         0         0         1
G10         1         1         0         2         0         0         0         0         0
    b000001.10 b000002.1 b000002.2 b000002.3 b000002.4 b000002.5
G01          0         2         0         0         0         0
G02          0         2         0         0         0         0
G03          0         2         0         0         0         0
G04          0         0         2         0         0         0
G05          0         2         0         0         0         0
G06          0         0         0         1         0         1
G07          0         0         2         0         0         0
G08          0         2         0         0         0         0
G09          1         0         2         0         0         0
G10          0         0         1         0         1         0
```

3 HAPLOTYPE BLOCKS BASED ON A FIXED NUMBER OF SNPS

For method `FIXED-SNP` with n =5, five consecutive SNPs are grouped into one haplotype block.

	***hb*1**					***hb*2**				
	$m01$	$m02$	$m03$	$m04$	$m05$	$m06$	$m07$	$m08$	$m09$	$m10$
$G01$	4/4	1/1	3/3	1/1	4/4	2/2	4/4	2/2	3/3	3/3
$G02$	2/4	−1/−1	3/3	1/1	3/3	2/4	4/4	2/2	3/3	3/3
$G03$	2/2	1/3	3/3	1/1	3/3	4/4	4/4	2/2	3/3	3/3
$G04$	2/2	1/1	3/3	1/1	4/4	2/2	2/2	4/4	2/2	4/4
$G05$	2/2	1/3	1/1	2/2	4/4	4/4	4/4	2/2	3/3	3/3
$G06$	2/2	3/3	1/1	2/2	3/3	4/4	2/4	2/2	2/2	3/3
$G07$	2/2	1/1	3/3	1/1	4/4	2/2	2/2	4/4	2/2	4/4
$G08$	4/4	3/3	1/1	2/2	3/3	4/4	4/4	2/2	3/3	3/3
$G09$	4/4	1/3	1/1	1/1	4/4	4/4	2/2	4/4	2/2	4/4
$G10$	2/4	3/3	1/1	2/2	3/3	4/4	2/4	2/4	2/2	4/4

Considering the sequence of markers within the two haplotype blocks, there are 12 variants for haplotype block $hb1$ and seven variants for haplotype block $hb2$.

Overview over distinct variants for haplotype blocks built with method `FIXED-SNP`, $n = 5$ (five SNPs per haplotype block):

Haplotype block	Variant	Sequence of SNP alleles
hb1	1	2;1;3;1;4
hb1	2	2;3;1;2;3
hb1	3	4;1;3;1;4
hb1	4	4;3;1;2;3
hb1	5	4;-1;3;1;3
hb1	6	2;1;1;2;4
hb1	7	2;3;1;2;4
hb1	8	2;-1;3;1;3
hb1	9	2;3;3;1;3
hb1	10	2;1;3;1;3
hb1	11	4;1;1;1;4
hb1	12	4;3;1;1;4
hb2	1	4;4;2;3;3
hb2	2	2;2;4;2;4
hb2	3	2;4;2;3;3
hb2	4	4;2;4;2;4
hb2	5	4;4;2;2;3
hb2	6	4;2;2;2;4
hb2	7	4;2;2;2;3

The following matrix assigns the haplotype blocks variants to the genotypes according to the SNP sequence that they show for the respective block. Note that haplotype blocks that contain missing SNP marker data are encoded as missing.

	$hb1$	$hb2$
$G01$	3/3	3/3
$G02$	−1/−1	3/1
$G03$	10/9	1/1
$G04$	1/1	2/2
$G05$	6/7	1/1
$G06$	2/2	7/5
$G07$	1/1	2/2
$G08$	4/4	1/1
$G09$	11/12	4/4
$G10$	2/4	6/4

Lastly, the re-parametrized design matrix for the haplotype blocks:

	$\boldsymbol{hb1}$										$\boldsymbol{hb2}$						
	$v01$	$v02$	$v03$	$v04$	$v06$	$v07$	$v09$	$v10$	$v11$	$v12$	$v01$	$v02$	$v03$	$v04$	$v05$	$v06$	$v07$
$G01$	0	0	2	0	0	0	0	0	0	0	0	0	2	0	0	0	0
$G02$	0	0	0	0	0	0	0	0	0	0	2	0	2	0	0	0	0
$G03$	0	0	0	0	0	0	1	1	0	0	2	0	0	0	0	0	0
$G04$	2	0	0	0	0	0	0	0	0	0	0	2	0	0	0	0	0
$G05$	0	0	0	0	1	1	0	0	0	0	2	0	0	0	0	0	0
$G06$	0	2	0	0	0	0	0	0	0	0	0	0	0	0	1	0	1
$G07$	2	0	0	0	0	0	0	0	0	0	0	2	0	0	0	0	0
$G08$	0	0	0	2	0	0	0	0	0	0	2	0	0	0	0	0	0
$G09$	0	0	0	0	0	0	0	0	1	1	0	0	0	2	0	0	0
$G10$	0	1	0	1	0	0	0	0	0	0	0	0	0	1	0	1	0
